# Genome-Wide Association Study of *d*-Amphetamine Response in Healthy Volunteers Identifies Putative Associations, Including Cadherin 13 (*CDH13*)

**DOI:** 10.1371/journal.pone.0042646

**Published:** 2012-08-28

**Authors:** Amy B. Hart, Barbara E. Engelhardt, Margaret C. Wardle, Greta Sokoloff, Matthew Stephens, Harriet de Wit, Abraham A. Palmer

**Affiliations:** 1 Department of Human Genetics, University of Chicago, Chicago, Illinois, United States of America; 2 Department of Computer Science, University of Chicago, Chicago, Illinois, United States of America; 3 Department of Psychiatry and Behavioral Neuroscience, University of Chicago, Chicago, Illinois, United States of America; 4 Department of Statistics, University of Chicago, Chicago, Illinois, United States of America; Johns Hopkins University, United States of America

## Abstract

Both the subjective response to *d*-amphetamine and the risk for amphetamine addiction are known to be heritable traits. Because subjective responses to drugs may predict drug addiction, identifying alleles that influence acute response may also provide insight into the genetic risk factors for drug abuse. We performed a Genome Wide Association Study (GWAS) for the subjective responses to amphetamine in 381 non-drug abusing healthy volunteers. Responses to amphetamine were measured using a double-blind, placebo-controlled, within-subjects design. We used sparse factor analysis to reduce the dimensionality of the data to ten factors. We identified several putative associations; the strongest was between a positive subjective drug-response factor and a SNP (rs3784943) in the 8^th^ intron of cadherin 13 (*CDH13*; *P* = 4.58×10^−8^), a gene previously associated with a number of psychiatric traits including methamphetamine dependence. Additionally, we observed a putative association between a factor representing the degree of positive affect at baseline and a SNP (rs472402) in the 1^st^ intron of steroid-5-alpha-reductase-α-polypeptide-1 (*SRD5A1*; *P* = 2.53×10^−7^), a gene whose protein product catalyzes the rate-limiting step in synthesis of the neurosteroid allopregnanolone. This SNP belongs to an LD-block that has been previously associated with the expression of *SRD5A1* and differences in SRD5A1 enzymatic activity. The purpose of this study was to begin to explore the genetic basis of subjective responses to stimulant drugs using a GWAS approach in a modestly sized sample. Our approach provides a case study for analysis of high-dimensional intermediate pharmacogenomic phenotypes, which may be more tractable than clinical diagnoses.

## Introduction

The subjective responses to amphetamine and the risk for amphetamine dependence are heritable traits [1], [2], [3]. It is hypothesized that the genetic variation underlying subjective drug responses may contribute to the risk of developing drug dependence [4], [5], [6], [7], [8], [9]. Previous genetic association studies of the response to amphetamine suggest a role for genetic sources of variability in acute drug effects, but have focused on candidate genes [1], [10], [11], [12], [13], [14], [15], [16], [17], [18], [19], [20]. Candidate gene studies are inherently limited in their ability to generate novel hypotheses compared with genome-wide association studies (GWAS). Here we report the results of the first GWAS for subjective response to acute administration of a drug of abuse in humans, using a laboratory-based, double-blind, placebo-controlled, within-subjects design to quantify subjective response to *d*-amphetamine in 381 healthy non-drug abusing participants, and testing for associations with 5.4 million (typed and imputed) single nucleotide polymorphisms (SNPs) across the genome.

Our study differs from most published GWAS in its use of complex multi-dimensional phenotypes rather than a binary diagnosis such as drug dependence or abuse. We obtained responses on three questionnaires, administered over six time points, in three sessions, after placebo or one of two doses of drug. Such multi-dimensional “intermediate phenotypes” have many advantages over binary diagnoses. Drug abuse is a heterogeneous phenotype that consists of a series of stages, each influenced by a variety of environmental and genetic factors [21]. In contrast, intermediate phenotypes can be measured under carefully controlled conditions, e.g., laboratory conditions, and may be directly linked to genetic variants and resulting from specific biological processes. For this reason, intermediate phenotypes are hypothesized to show stronger genetic associations than binary diagnoses, potentially allowing for smaller sample sizes [22]. For example, a GWAS of electroencephalogram (EEG), which is an intermediate phenotype for brain-related clinical endpoints, reported genome-wide significant associations using just 322 participants [23]. Furthermore, acute responses to pharmacological perturbations often yield alleles of relatively large effects [24]. Association studies of the response to cisplatin [25], warfarin [26], nortriptyline [27], radiation therapy [28], pegylated interferon and ribavirin [29], and interferon-β [30] have all identified genome-wide significant associations using small samples sizes.

Despite the potential advantages noted above, high-dimensional intermediate phenotypes pose considerable analytic challenges. Here we describe the results of an innovative strategy for analyzing these data, which includes methods to normalize phenotypes and application of sparse factor analysis (SFA) [31], [32] to provide interpretable summary phenotypes. Our approach provides a model for the analysis of other high-dimensional intermediate phenotypes in the context of genetic association studies.

## Materials and Methods

### Participants

Healthy young adults (N = 381 final sample; 200 male, 181 female) aged 18–35 years old were recruited and screened through a physical examination, electrocardiogram, modified Structured Clinical Interview for DSM-IV, psychiatric symptom checklist (SCL90) and self-reported health and drug use history. Exclusion criteria were: past year Axis I Disorder, history of mania or psychosis, less than a high-school education, smoking more than ten cigarettes per week, drinking more than three cups of coffee per day, lack of English fluency, or medical contraindication to amphetamine administration. Participants were inexperienced stimulant users: only 24% reported any previous use of stimulants and less than 2% reported 50 or more lifetime uses. Participants were asked to abstain from drugs and alcohol for 24 hours, nicotine for 12 hours and to fast for 12 hours prior to each session. At the start of each session, participants provided urine (ToxCup, Branan Medical Corporation, Irvine, CA, USA) and breath samples (Alcosensor III, Intoximeters Inc., St. Louis, MO, USA; piCO+ Smokerlyzer, Bedfont, Rochester, UK) to confirm drug, alcohol and nicotine abstinence, and female participants were tested for pregnancy.

### Phenotyping

Participants completed three randomized 4-hour study sessions, separated by at least 72 hours, during which they received placebo or *d*-amphetamine (10 or 20 mg) under double-blind conditions. They completed questionnaires measuring subjective effects before and 30, 60, 90, 150 and 180 minutes after ingesting the capsule; heart rate and blood pressure were also measured at these times; these procedures have been described previously [1] (also see Supporting Information S1). Subjective responses were measured using the Profile of Mood States (POMS) [33], the Drug Effects Questionnaire (DEQ) [34], and the Addiction Research Center Inventory (ARCI) [35], [36]. Thus, 22 phenotypes were obtained in four categories: 1) physiological: systolic and diastolic blood pressure, heart rate, 2) POMS scales: Friendliness, Anxiety, Depression, Fatigue, Anger, Elation, Confusion, Vigor, 3) DEQ scales: Feel Drug, Feel High, Want More, Like Drug, Dislike Drug, and 4) ARCI scales: Amphetamine, Benzedrine Group (BG), Marijuana, Lysergic Acid (LSD), Morphine-Benzedrine Group (MBG) and Pentobarbitol-Chlorpromazine-Alcohol Group (PCAG). Each of these 22 phenotypes was measured at six time points during three sessions (placebo, 10 and 20 mg), totaling 396 phenotypic values for each participant. This study was approved by the Institutional Review Board of The University of Chicago and was carried out in accordance with the Helsinki Declaration of 1975. Written informed consent was obtained from all participants.

### Data summarization and dimension reduction

First we imputed missing phenotype values using probabilistic principal components analysis [37], [38] with five principal components, using the R [39] package ppca [40]. This approach exploits correlations among phenotypes to impute missing values. In the final dataset 98.74% of phenotype values were observed and 1.26% were imputed.

In addition to the primary outcome phenotypes, several demographic measures were collected (Table S1). We examined the correlations between these demographic measures and the outcome phenotypes. We observed strong correlations between age, gender, and BMI and multiple response measures (Figure S1). Notably, we did not see strong correlations between current or past drug use and any of the response measurements in this sample of light drug users. For each outcome phenotype we derived a corrected phenotype by taking the residuals of a linear regression model, using age, sex and BMI as covariates.

**Figure 1:**
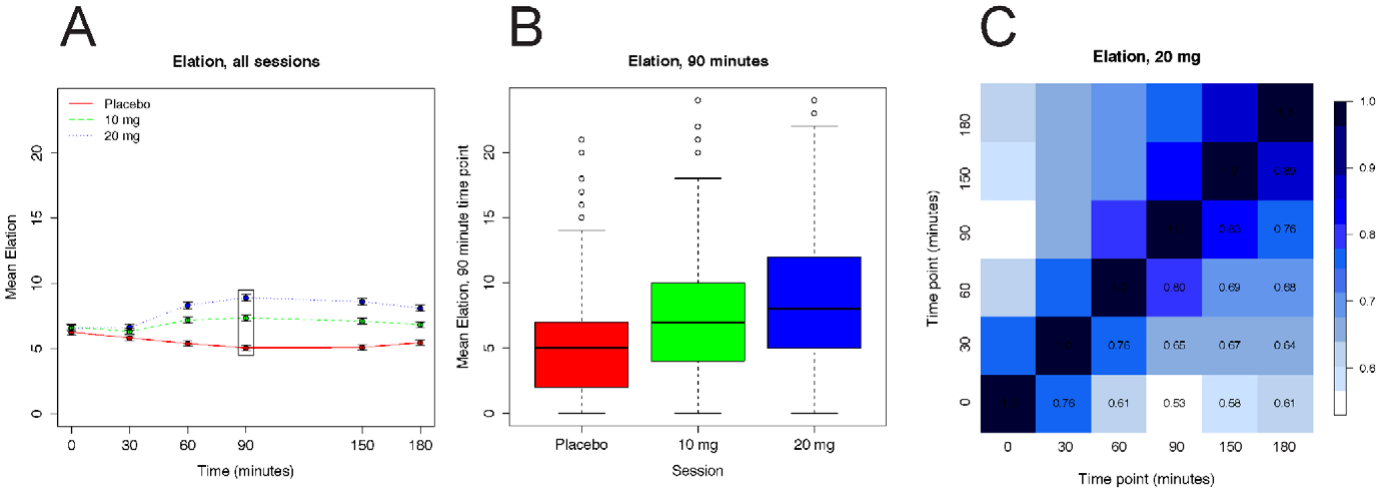
Elation (POMS) example phenotype. Panel A shows the mean (SEM) scores on the POMS Elation scale at each time point before and after administration of placebo or *d*-amphetamine (10 and 20 mg). The box identifies the values shown in greater detail in Panel B. Panel B shows a boxplot of the Elation phenotype at 90 minutes after placebo, 10 or 20 mg *d*-amphetamine sessions. Panel C shows the Pearson correlation coefficient for the Elation phenotypes at all six time points for the 20 mg session: darker blue indicates high correlation and white indicates low correlation.

Amphetamine treatment significantly affected most of the phenotypes (Table S2). Exploratory data analyses of each phenotype using both individual data and the mean values showed the expected drug effects across the entire sample (Figure 1A), but we observed substantial variability at individual data points (Figure 1B). Average phenotypic values obtained at baseline, before the drug was administered (0 min), were similar across the three sessions. Average phenotypic values obtained at the second time point (30 min) typically showed minor drug effects, whereas phenotypic values at each of the remaining time points changed in a dose-dependent manner. Although the mean values at the last four time points were generally consistent with the expected time course (increasing to a peak, and then slowly decreasing) these time-points were highly correlated (Figure 1C) and the differences among these means were generally small compared with the difference from the mean at the 0 time point, particularly in the non-placebo sessions. Therefore, we summarized the six measurements for each phenotype in each session, for each individual, using two numbers: (i) the first time point (0 min), which we refer to as the *baseline* measure; and (ii) the mean of the last four time points (60, 90, 150, 180 min) controlling for the first time point (0 min), which we refer to as the *response* measure. Note that, by construction, these two summary values are uncorrelated.

An alternative that we considered but rejected was to combine the first two time points to form the baseline measure. Factor loadings were quite similar using this approach; however, we observed some evidence of early drug effects for some measures in a subset of participants at the 30 minute time point, and thus we chose to omit the 30 minute time point to avoid controlling for drug response.

The above process yielded a baseline and response measure for each of the 22 phenotypes in each session (placebo, 10 mg, and 20 mg). Next we sought to combine information across sessions and phenotypes to yield a small number of phenotypes that efficiently captured the primary patterns in the whole data; this process is commonly referred to as *dimension reduction*. Combining data across correlated measurements reduces environmental noise and measurement error. We chose to perform dimension reduction using sparse factor analysis (SFA) [31], which is a variant of factor analysis that attempts to improve interpretability of factors (summary measures) by encouraging each factor to be a summary of a small subset of all data points. Because SFA is based on a normal model, we transformed each baseline and response measure to follow the standard normal distribution (using a quantile-quantile transformation) and applied SFA to these transformed data. After experimenting with different numbers of factors we chose eleven factors as providing the most reproducible and interpretable results, yielding an 11 by 381 matrix of measurements. Of the 11 factors identified, one was strongly correlated with age, and so we disregarded that factor for all subsequent analysis. Each of the remaining factors (F1–F10, Figure S2) captured distinct, and generally interpretable, features of the data. For example, F1 almost exclusively consists of measurements of response from the 10 mg session, and so we refer to this factor as “10 mg response”. Factor F2 is primarily a function of baseline measures of positive affect from all three sessions (e.g., positive loadings on Friendliness, Elation, and Vigor; negative loadings on Anxiety and Confusion), and we refer to this factor as “positive affect at baseline” (Table S3). Although some factors might be considered better candidates for being influenced by genetic factors than others, we conducted association mapping on all 10 factors since a non-genetic factor should produce null results. The interpretability of factors obtained using SFA compared favorably to alternative dimension reduction techniques such as PCA (see Supporting Information S1 for further discussion).

### Genotyping and quality control

DNA was extracted from blood at the General Clinical Research Center at the University of Chicago. Genotyping was performed using the Affymetrix 6.0 array at the Functional Genomics Core Facility (Vanderbilt University, Memphis, TN, USA). The arrays were passed through the Affymetrix apt-geno-qc package, and DM call rate, contrast QC metric, and genotypic gender were computed. Genotypes were called with the Birdseed [41] and CRLMM [42] algorithms. We imputed missing and non-genotyped SNPs with the IMPUTE2 software package [43], using the 1000 Genomes [44] and HapMap 3 [45] genotypes as reference panels. Imputation brought the total number of SNPs to 7,573,542 SNPs per individual. We removed SNPs that had a MAF<0.05 in our sample because they had low power to identify associations; this left each individual with 5,476,100 SNPs. The SNPs presented in the Results section were re-genotyped using an Applied Biosystems TaqMan® SNP Genotyping Assay (Table S4).

### Association mapping

We performed association mapping separately for each of the ten factors in 381 individuals. First, to reduce potential problems due to non-normality, each factor was quantile-quantile transformed to a standard normal distribution (*quantile normalized*). We then corrected for potential population stratification by controlling each factor for the first two principal components computed by applying SmartPCA [46] to the genotype data (Figure S3). Finally, these residuals were again quantile normalized to form the final 10 factors that were used for association testing.

We tested for association of each factor with each SNP by linear regression of the factor against SNP genotype. For imputed SNPs and missing genotypes we used the posterior mean genotype [47]. For each SNP and factor we performed both frequentist and Bayesian tests of association: we obtained *P*-values using SNPTest [48] with the parameters: [-frequentist 1 -method expected -use_raw_phenotypes] and Bayes Factors (BF) using BIMBAM [47], [49] using the default parameters that average over four different prior probabilities on effect size.

*Post hoc* analyses were carried out to determine the effect of the two most significant SNPs on individual phenotypes; 3×3×6 repeated measures ANOVAs (SPSS 17.0) were conducted with genotype as the grouping factor and dose and time as the two within-subjects factors; age, sex, BMI, and the first two principal components from SmartPCA were included as covariates.

Because 325 of the 381 participants were Caucasians (based on both self-report and clustering with SmartPCA), we also performed association mapping on this Caucasian-only subset for those SNPs highlighted in the Results section without controlling for population stratification (see Supporting Information S1 for additional details).

## Results

We performed a GWAS on 381 participants for ten phenotypes at 5,476,100 SNPs; results are shown in Table S4. Across the 10 factors we identified associations whose significance approached or exceeded 5×10^−8^, which is often used as the threshold for genome-wide significance. No single test produced a *P*-value low enough for us to consider it incontrovertible. Nonetheless, the two most significant associations (one with a baseline factor, and one with a response factor) involved genes that, on the basis of prior biological evidence, are good candidates for being associated with these phenotypes. We view these results as generating credible hypotheses that can be evaluated in future studies.

**Figure 2:**
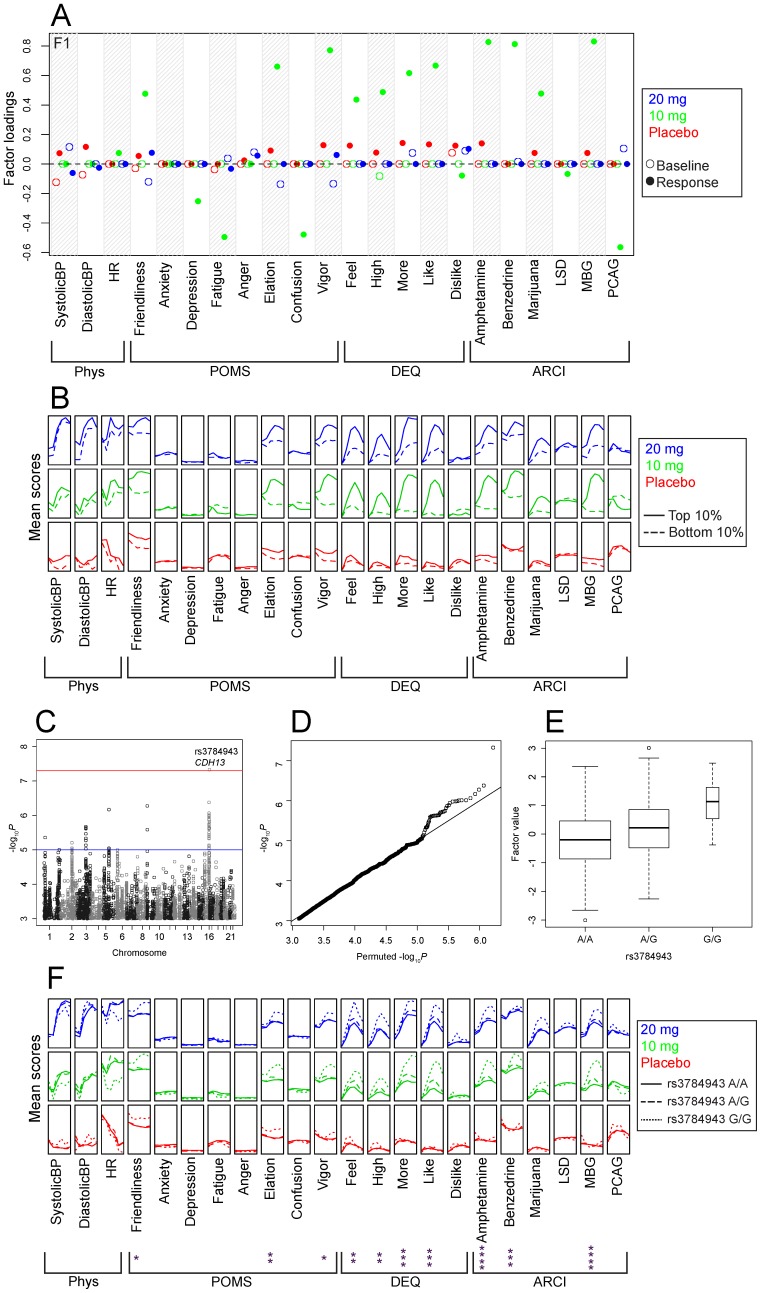
Potential association of rs3784943 (*CDH13*) with an amphetamine response factor. Panel A shows the factor loadings for factor F1; abbreviations of outcome measures are defined in the text. Panel B shows the raw phenotype scores as a function of dose (denoted by color coded vertically stacked panels) and time (x-axis within each panel) for individuals in the upper and lower deciles of this factor. Panel C shows the association between factor F1 and each SNP (expressed as −log_10_ *P*), the most significant association was on chromosome 16 at rs3784943 (*P* = 4.58×10^−8^). The red horizontal line indicates 5×10^−8^, which is often used as a threshold for significance. The blue horizontal line indicates 1×10^−5^, which could be considered a threshold for suggestive evidence. Panel D shows a Q-Q plot of observed −log_10_ *P* versus the average −log_10_ *P* from ten random permutations. Panel E shows a boxplot of the values for factor F1 stratified by genotype. The width of each box corresponds to number of observations at the corresponding genotype. Panel F shows the raw phenotype scores stratified by genotypes as a function of dose and time. Asterisks indicate a significant Drug×time×genotype interaction in a 3×3×6 repeated measures ANOVA using age, sex, BMI, and the first two principal components as covariates. * *P*≤0.05; ** *P*≤0.01; *** *P*≤0.0001; **** *P*≤10^−4^.

We identified a potential association between rs3784943 and factor F1, which we labeled “10 mg response” (*P* = 4.58×10^−8^; log_10_BF = 5.26). The frequency of this SNP did not show marked differences among populations in 1000 Genomes, and an analysis of the Caucasian-only subset yielded consistent results (*P* = 6.22×10^−7^; log_10_BF = 3.53). The factor loadings show that this factor consists primarily of responses in participants in the 10 mg session. Friendliness, Elation, Vigor, Feel, High, More, Like, Amphetamine, Benzedrine, Marijuana and MBG have positive loadings on this factor, while Depression, Fatigue, Confusion and PCAG have negative loadings (Figure 2A). Examination of the mean values of the raw phenotypic data for individuals in the upper and lower deciles of this factor show that, although the 20 mg session values did not contribute to the factor through the loadings, there is substantial correlation with a positive response to 20 mg (Figure 2B). Rs3784943 falls within the 8^th^ intron of the gene cadherin 13 (*CDH13*) which codes for a cell adhesion molecule that is highly expressed in the brain [50]. The minor allele frequency of this SNP in our sample was 22%; the HWE *P*-value was 0.231. The genome-wide *P*-values for this factor are shown in Figure 2C, and their distribution compared with its expectation under the null (obtained via permutations) is shown in Figure 2D. Differences in factor F1 for the three genotypes are shown in Figure 2E. We also examined the unprocessed phenotypic data stratified by genotype at this SNP and ran repeated measures ANOVAs to determine which individual phenotypes were associated with rs3784943 (Figure 2F). When performing dimension reduction on a complex dataset, it is critical to examine the impact of the SNP on the high-dimensional phenotypes rather than only the effect size on the one-dimensional factor.

**Figure 3:**
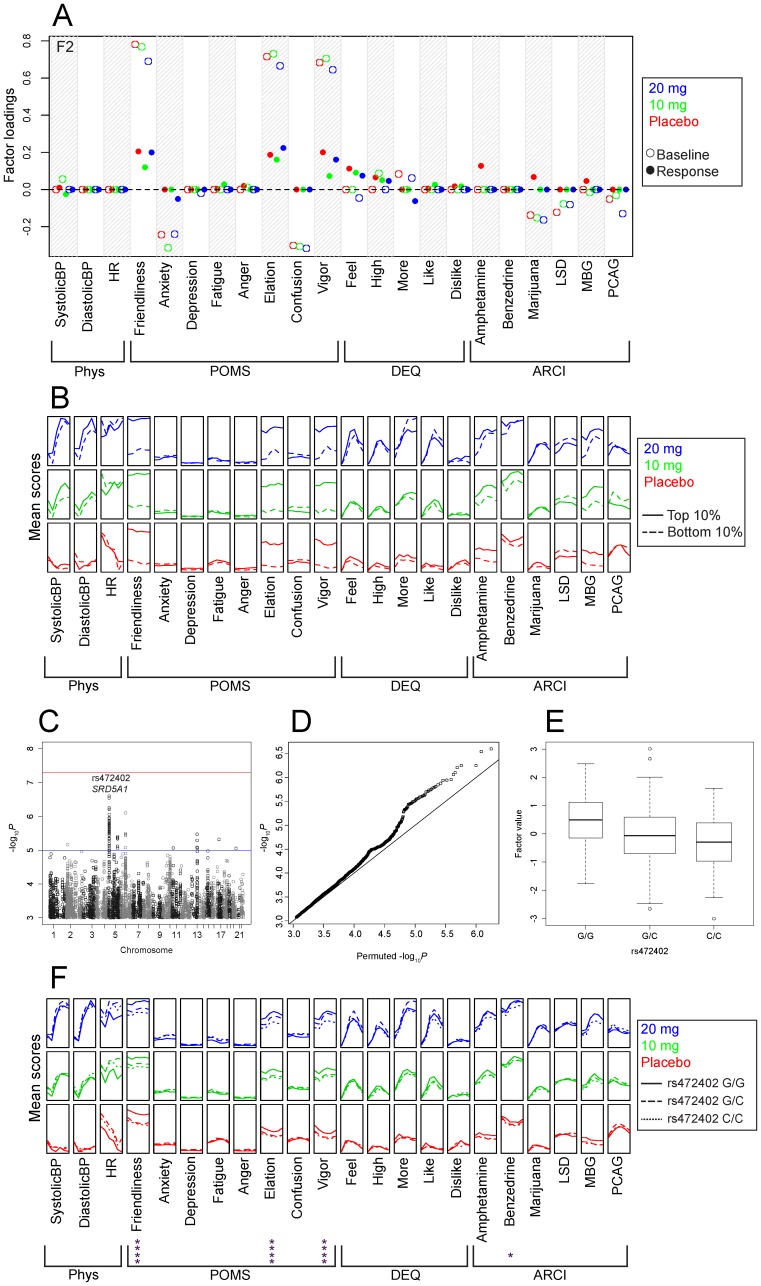
Potential association of rs472402 (*SRD5A1*) with the positive affect baseline factor. Panel A shows the factor loadings for factor F2; abbreviations of outcome measures are defined in the text. Panel B shows the raw phenotype scores as a function of dose (denoted by color coded vertically stacked panels) and time (x-axis within each panel) for individuals in the upper and lower deciles of this factor. Panel C shows the association between factor F1 and each SNP (expressed as −log_10_ *P*), the most significant association was on chromosome 5 at rs472402 (*P* = 2.53×10^−7^). The red horizontal line indicates 5×10^−8^, which is often used as a threshold for significance. The blue horizontal line indicates 1×10^−5^, which could be considered a threshold for suggestive evidence. Panel D shows a Q-Q plot of observed −log_10_ *P* versus the average −log_10_ *P* from ten random permutations. Panel E shows a boxplot of the values for factor F1 stratified by genotype. The width of each box corresponds to number of observations at the corresponding genotype. Panel F shows the raw phenotype scores stratified by genotypes as a function of dose and time. Asterisks indicate a significant main effect of genotype in a 3×3×6 repeated measures ANOVA using age, sex, BMI, and the first two principal components as covariates. * *P*≤0.05; **** *P*≤10^−4^.

We also identified a potential association between rs472402 and factor F2, which we labeled “positive affect at baseline” (*P* = 2.53×10^−7^; log_10_BF = 4.33). Similar to the previous example, this SNP did not show marked differences among populations in 1000 Genomes, and an analysis of the Caucasian-only subset yielded consistent results (*P* = 4.62×10^−5^; log_10_BF = 2.47). Several other SNPs in strong linkage disequilibrium (LD) with rs472402 (r^2^ = 0.77–0.98) were also associated with the baseline positive affect factor (see Table S5). Friendliness, Elation, and Vigor baseline values for all three sessions have substantial positive loadings on this factor, while Anxiety and Confusion baseline values have substantial negative loadings (Figure 3A). Examination of the mean values of the raw phenotypic data for individuals in the upper and lower deciles of this factor show that Friendliness, Elation, and Vigor were markedly different across sessions and time points (Figure 3B). Rs472402 falls within the first intron of the gene *SRD5A1*, which codes for an enzyme that converts progesterone to allopregnanolone, among other functions [51]. The minor allele frequency of this SNP in our sample was 48.6%; the HWE *P*-value was 0.305. The genome-wide *P*-values for this factor are shown in Figure 3C, and their distribution compared with the *P*-values under the null (obtained via permutations) is shown in Figure 3D. Differences in the factor values for the three genotypes are shown in Figure 3E. The unprocessed phenotypic data stratified by genotype at this SNP is shown in Figure 3F. Repeated measures ANOVAs were run to determine which individual phenotypes were associated with rs472402.

## Discussion

We performed a GWAS for subjective response to *d*-amphetamine in a small sample of healthy, non-drug abusing participants who were phenotyped using a laboratory-based, double-blind, placebo-controlled, within-subjects design, using 5.4 million genotyped or imputed SNPs. Phenotypic data were summarized using SFA, which yielded ten generally interpretable factors that represented both drug-independent and drug response phenotypes. We identified a SNP in the *CDH13* gene (Figure 2) that was associated with the degree of positive response to amphetamine. We also identified a SNP in the *SRD5A1* gene (Figure 3) that was potentially associated with the degree of positive affect of the participants, independent of dose.

Our strongest genetic association with a drug response factor was between rs3784943 and factor F1 (Figure 2). This SNP is in the 8^th^ intron of *CDH13*, which is highly expressed in multiple brain regions [50]. *CDH13* has been implicated in drug-abuse related phenotypes by multiple studies from the same group that aggregate evidence at the gene level; specifically, methamphetamine dependence [52], alcohol dependence [53], [54], nicotine dependence [55], [56], successful smoking cessation [57], polysubstance dependence [58], addiction vulnerability [59], and comorbid depression and alcohol dependence [60]. In addition, a meta-analysis of cigarettes smoked per day also identified multiple SNPs in *CDH13* [61]. *CDH13* has also been implicated in attention deficit hyperactivity disorder (ADHD) at multiple SNPs [62], including rs11646411 [63] and rs6565113 [64]. A meta-analysis of several GWAS for ADHD identified rs8045006 [65]. Finally, a meta-analysis of linkage scans for ADHD identified a genomic region containing *CDH13* (16q21–16q24) [66]. Based on these results, a candidate gene study of *CDH13* and ADHD was performed that found a gene-wide significant association at one SNP (rs11150556) [67]. In addition, *CDH13* has also been implicated by GWAS in depression (rs10514585) [68], extraversion (rs4783307, rs8056579) [69], agreeableness (rs9940706) [69] and response to antipsychotic therapy (rs17216786) [70], as well as in a meta-analysis for extraversion (rs8057458) [71]. Variants in *CDH13* have also been significantly associated with adiponectin levels in two GWAS [72], [73]. None of the SNPs implicated by the above studies were strongly associated with our factors, nor were any of the above-mentioned SNPs in strong LD with rs3784943. Thus, while this gene has attracted significant attention, particularly in the psychiatric genetics literature, these studies have not converged on a single genetic regulator or putative mechanism of regulation for this gene for possibly corresponding phenotypic effects. Intriguingly, it has recently been reported that *CDH13* knockout mice show decreases in conditioned place preference to cocaine (J. Drgonova, SFN abstract #871.11/D64), which is highly consistent with a difference in the subjective response to amphetamine that we observed.

The association between *CDH13* and sensitivity to the subjective effects of amphetamine may provide insight into the mechanism by which an allele influences the risk for drug dependence. Drug dependence develops through many stages, including initial experimentation with drugs, continued use, dependence, withdrawal and relapse [74]. Genetic variants might influence risk by impacting one or more of these stages [75]. Whereas a genetic association with the diagnosis of drug dependence provides little insight into which stage is under genetic control, our intermediate phenotype approach suggests that rs3784943 affects magnitude of the initial subjective response to the drug. This would be expected to influence an early stage in the addiction process in which early experimentation with drugs progresses to more frequent drug use. Further studies are needed to assess whether this SNP might also influence the subjective response to other drugs of abuse.

Our strongest association with a baseline factor was between rs472402 and factor F2 (Figure 3). This SNP is in the first intron of *SRD5A1*, which is expressed in the brain [76]. The protein product of this gene catalyzes the rate-limiting step in the production of the neurosteroid allopregnanolone [77], which is a GABA_A_ agonist. Allopregnanolone has been shown to have anxiolytic effects in animals [78] and progesterone administration, perhaps because it is converted into allopregnanolone, has been shown to elicit mild sedative effects in humans [79], [80], [81]; these are broadly similar to the phenotypes that are associated with this SNP (Figure 2F). Another SNP (rs248797; Table S5) that is in strong LD with rs472402 (r^2^ = 0.73) has been identified as a *cis*-eQTL for the *SRD5A1* gene in human monocytes [82] (*P* = 6×10^−19^) and is also in moderate LD (r^2^ = 0.3–0.7) with several SNPs that have been identified as *cis*-eQTLs for *SRD5A1* in post-mortem samples from cerebellum and parietal cortex [83]. Rs472402 is also in strong LD with rs248793 (r^2^ = 0.89), which has been associated with differences in SRD5A1 activity [84] and with risk for alcohol dependence [85]. Finally, *SRD5A1* has also been suggested as a positional candidate gene based on a linkage study for cocaine dependence and major depressive episode [86]. Taken together these results suggest that SNPs in *SRD5A1* could influence subjective positive affect by modulating both expression and enzymatic activity of *SRD5A1* and thereby altering allopregnanolone levels in the brain.

While techniques that aggregate information using dimension reduction are attractive, the results must be both interpretable and biologically meaningful. Our results here illustrate how SFA can yield more interpretable data summaries than other methods, such as PCA, facilitating the systematic aggregation of a large collection of phenotypic information without sacrificing interpretability. Regardless of the dimension reduction technique used to identify putative associations, it is important to examine the raw phenotypes to determine how a given SNP correlates with the underlying phenotypic data (Figure 2F and Figure 3F). In both of the potential associations presented in this study, the phenotypes that loaded onto the factor overlapped well with, but were not identical to, the phenotypes that were influenced by the identified SNPs (Figure 2A and Figure 3A).

This study has several strengths and limitations. One limitation is that we used a relatively small number of participants to test a large number of putative predictors (genotypes). Despite the small sample, there is credible evidence that intermediate pharmacogenomic phenotypes might be more likely to have larger effect alleles [24]. Another potential limitation of this study was the use of participants that were not heavy drug abusers and thus might not have been at the highest genetic risk for developing drug abuse. Although our study was based on the idea that genetic variability in a population of healthy young adults is representative of the larger population, it is possible that our ascertainment procedures excluded relevant genetic variants. Similarly, we chose modest doses that were administered orally, whereas drug users typically ingest higher doses with faster routes of administration (intranasal, intravenous, or inhalation). Lastly, differences in drug metabolism may constitute an uncontrolled source of variability. Reducing the complex phenotype data to a small number of baseline and response factors is a strength of our study because baseline differences might otherwise confound our measurement of drug response. We chose to compute associations for factors representing both drug-independent and drug-dependent phenotypes because we believed that genetic associations with either baseline or response measurements are potentially interesting, although of course our primary motivation for the study was to study drug response.

While intermediate phenotypes have many advantages, a major limitation is that replication samples are seldom available. We were unable to identify any suitable replication dataset for the association between the SNP (rs3784943) in *CDH13* and positive subjective response. Replication of this result was challenging because a suitable study would have included administration of a stimulant drug, under similar conditions and corresponding dosage, and would have had to collect the same or related measurements of subjective response. In addition, the associated SNP had a fairly low minor allele frequency and only a moderate effect on the phenotype, so a relatively large dataset would be needed to have sufficient power for replication. Alternatively, our results can be viewed as replicating prior associations with *CDH13*, however the lack of convergence on a single SNP or haplotype across studies is a cause for concern. Because of these limitations, the results from *Cdh13* knockout mice and other biologically based studies of *CDH13* function may be the best route for further investigation and replication of our results.

Replication of the association between the SNP (rs472402) in *SRD5A1* and baseline positive affect appeared to be a more tractable problem because multiple GWAS datasets that include measurements of personality traits are available. Although our questionnaires were designed to detect differences in mood state, the effect persisted regardless of session or time point, which is consistent with a difference in trait independent of drug or session effects. We examined SNPs in strong LD with rs472402 in subjects from the National Institutes of Health/National Institute of Mental Health (NIH/NIMH) bipolar genetics studies with personality trait data consisting of the ZKPQ (n = 1007) and TCI (n = 944) subscales [87]; none of our analyses provided evidence of replication. However, the differences in the phenotypes and the study design were substantial between these studies and our own; this may indicate a poor fit for replication. We also considered the baseline sessions from smaller datasets collected in our lab using similar methodology but testing different drugs, however none of these datasets showed convincing evidence for replication. Taken together these results do not support the association between rs472402 and baseline positive affect.

In conclusion we have performed the first GWAS of the subjective response to a drug of abuse and identified two interesting associations. *CDH13* has previously been associated with a number of substance abuse and other psychiatric phenotypes and is also supported by data from knock-out mice. Replication in independent datasets will be important to establish the role of *CDH13* in drug addiction, and to determine the extent to which initial drug responses are related to the etiology of addiction. *SRD5A1* is similarly supported by prior evidence including corroborating genetic studies, gene expression and enzymatic activity data, but the lack of replication of this result is a cause for concern. We were motivated to perform this study using a relatively modest sample size because we believed that intermediate pharmacogenomic phenotypes might be influenced by alleles that contribute a larger fraction of the genetic variance. This study reflects both the utility and challenges of such phenotypes for discovery and enrichment of our understanding of complex psychiatric constructs such as drug abuse.

## Supporting Information

Figure S1 **Correlation between putative demographic and other covariates and raw phenotype data.** The putative covariates are on the x-axis; the raw data for each subscale for each time point and session are on the y-axis. Abbreviations on the x-axis are as follows: body mass index (BMI), alcoholic drinks per week (AlcWeek), cigarettes smoked per week (CigWeek), cups of caffeinated beverages per week (CaffWeek), times smoking marijuana per month (MarijMonth), ever used sedatives (SedEver), ever used stimulants (StimEver), ever used opiates (OpiatEver), ever used hallucinogens (HallEver), ever used inhalants (InhalEver), ever used marijuana (MarijEver). Abbreviations on the y-axis are defined in the text with the following exceptions: Physiological phenotypes (Phys), heart rate (HR), diastolic blood pressure (DiastolicBP), systolic blood pressure (SystolicBP). The Pearson correlation coefficient is indicated according to the scale bar on the right. Based on these data, Age, Gender and BMI were regressed from the phenotypic data.
(PDF)

Figure S2 **Factor loadings and GWAS results for all 10 factors.** Panel A shows the factor loadings; abbreviations of outcome measures are defined in the text. Percentage of variance explained (PVE), or the contribution to the variance in the survey data by each factor, is shown in the top left hand corner. Note that the PVE does not sum, because there is correlation among the factors. Panel B shows the raw phenotype scores as a function of dose (denoted by color coded vertically stacked panels) and time (x-axis within each panel) for individuals in the upper and lower deciles of this factor. Panel C shows the Manhattan plot of observed −log_10_ *P* for each chromosome; the red horizontal line indicates 5×10^−8^, which is often used as a threshold for significance. The blue horizontal line indicates 1×10^−5^, which could be considered a threshold for suggestive evidence. Panel D shows a Q-Q plot of observed −log_10_ *P* versus the average −log_10_ *P* from then random permutations. Factor descriptions are as follows (defined by Panels A and B): F1) responses during the 10 mg and 20 mg sessions, with highest scores on the 10 mg session; F2) positive affect at baseline for all three sessions; F3) responses during the 10 mg and 20 mg sessions, with highest scores on the 20 mg session; F4) responses during the 10 mg and 20 mg sessions; F5) negative affect at baseline for all three sessions; F6) blood pressure baseline measurements for all sessions; F7) responses primarily during the placebo session; F8) baseline measurements for the placebo session; F9) baseline measurements for the 10 mg session; F10) baseline measurements for the 20 mg session.
(PDF)

Figure S3 **Summary of genotyping quality control results.** Panel A shows observed HWE *P*-values plotted against expected *P*-values as computed by PLINK in the Caucasian-only sample. A cutoff of 10^−4^ was used and 149 SNPs were removed. Panel B shows the first two genetic principal components computed with SmartPCA on the full sample of 381 individuals. Individuals are color coded according to self-reported ancestry.
(PDF)

Table S1 **Demographic characteristics of the participant sample.** Mean values are expressed as mean ± SEM.
(DOC)

Table S2 **Amphetamine effects on individual scales.** POMS is Profile of Mood States questionnaire; DEQ is Drug Effect Questionnaire; ARCI is Addiction Research Center Inventory questionnaire. LSD is Lysergic acid; MBG is Morphine-Benzedrine Group; PCAG is Pentobarbitol-Chlorpromazine-Alcohol Group.
(DOC)

Table S3 **Description of factors obtained from Sparse Factor Analysis.** Interpretations based on factor loading plots (Figure S2A) and decile plots (Figure S2B) are given. PVE refers to percentage of variance explained by each individual factor.
(DOC)

Table S4 **Association results for all SNPs with** ***P*** **<1×10^−5^.** Factor designations are given in column 1: F1) responses during the 10 mg and 20 mg sessions, with highest scores on the 10 mg session; F2) positive affect at baseline for all three sessions; F3) responses during the 10 mg and 20 mg sessions, with highest scores on the 20 mg session; F4) responses during the 10 mg and 20 mg sessions; F5) negative affect at baseline for all three sessions; F6) blood pressure baseline measurements for all sessions; F7) responses primarily during the placebo session; F8) baseline measurements for the placebo session; F9) baseline measurements for the 10 mg session; F10) baseline measurements for the 20 mg session. *P*-values and log_10_BF are shown for SNPs highlighted in the Results section.
(XLS)

Table S5 **Association of a linkage disequilibrium block within** ***SRD5A1*** **and F2, the positive affect at baseline factor.** The post-re-genotyping association *P*-value, log_10_BF, and HWE *P*-value are given. SNPs with direct eQTL evidence in the eqtl.uchicago.edu database are noted.
(XLS)

Supporting Information S1 **Combined Supporting Materials and Methods and Supporting Results file.** Additional details of the methods are provided in the Supporting Materials and Methods section. Alternative dimensionality methods are detailed in the Supporting Materials and Methods and Results sections.
(DOC)
