# Supplementary figures and images for: Genome-Wide Association Study of d-Amphetamine Response in Healthy Volunteers Identifies Putative Associations, Including Cadherin 13 (CDH13)
Source: PLoS One. 2012 Aug 28;7(8):e42646. doi: 10.1371/journal.pone.0042646 (PMC3429486; doi:10.1371/journal.pone.0042646)

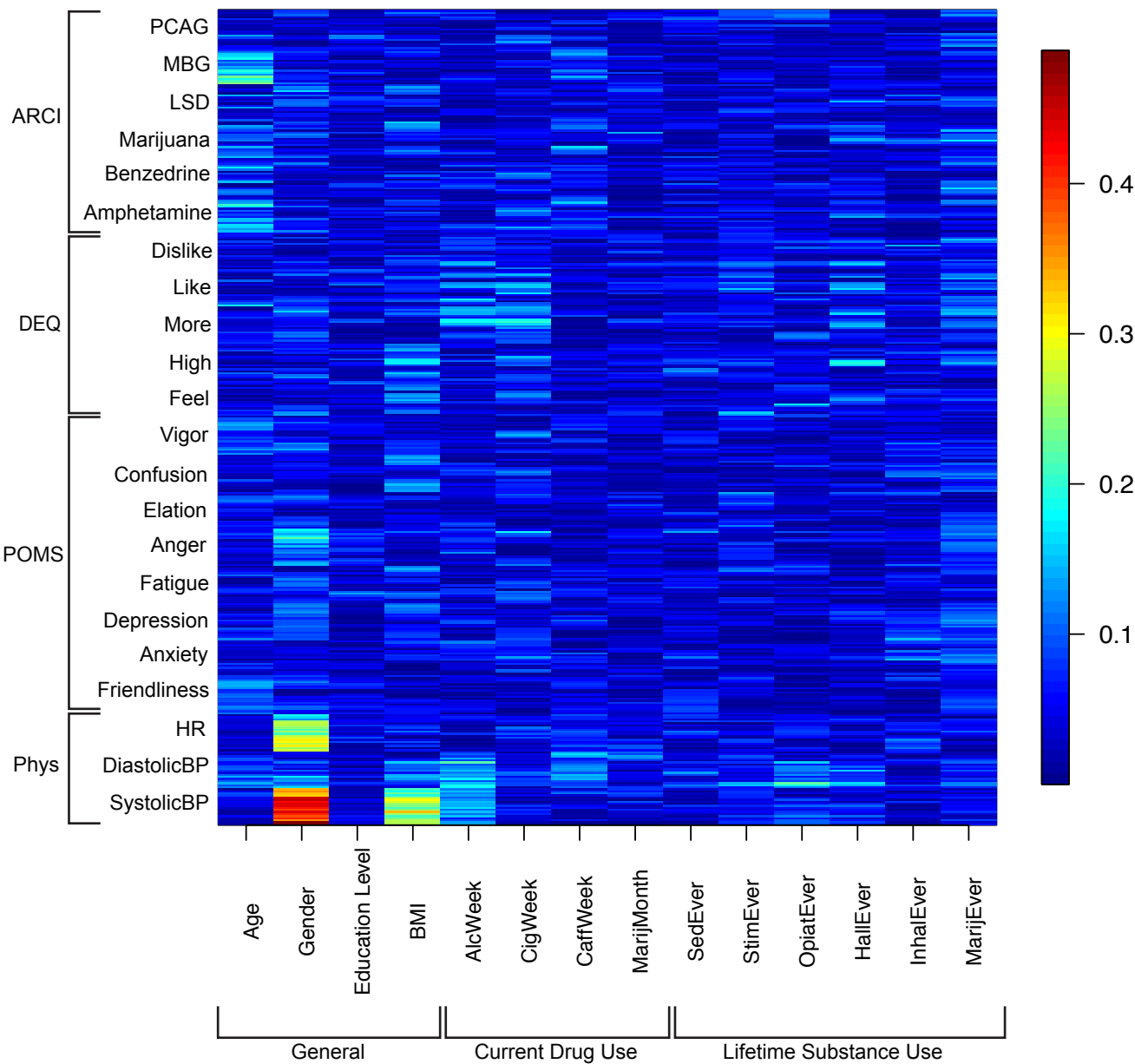

Supplement: Figure S1 — Correlation between putative demographic and other covariates and raw phenotype data. The putative covariates are on the x-axis; the raw data for each subscale for each time point and session are on the y-axis. Abbreviations on the x-axis are as follows: body mass index (BMI), alcoholic drinks per week (AlcWeek), cigarettes smoked per week (CigWeek), cups of caffeinated beverages per week (CaffWeek), times smoking marijuana per month (MarijMonth), ever used sedatives (SedEver), ever used stimulants (StimEver), ever used opiates (OpiatEver), ever used hallucinogens (HallEver), ever used inhalants (InhalEver), ever used marijuana (MarijEver). Abbreviations on the y-axis are defined in the text with the following exceptions: Physiological phenotypes (Phys), heart rate (HR), diastolic blood pressure (DiastolicBP), systolic blood pressure (SystolicBP). The Pearson correlation coefficient is indicated according to the scale bar on the right. Based on these data, Age, Gender and BMI were regressed from the phenotypic data. (PDF) [file pone.0042646.s001.pdf]

A

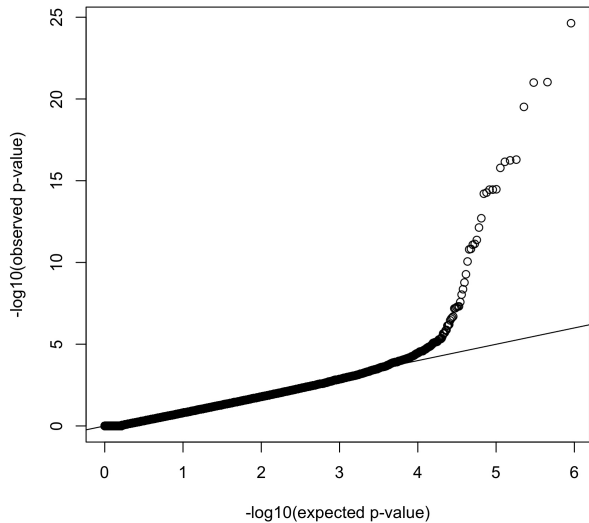

B

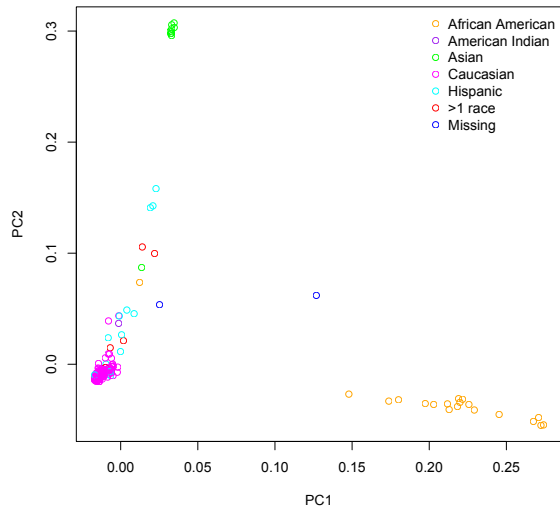

Supplement: Figure S3 — Summary of genotyping quality control results. Panel A shows observed HWE P-values plotted against expected P-values as computed by PLINK in the Caucasian-only sample. A cutoff of 10−4 was used and 149 SNPs were removed. Panel B shows the first two genetic principal components computed with SmartPCA on the full sample of 381 individuals. Individuals are color coded according to self-reported ancestry. (PDF) [file pone.0042646.s003.pdf]
