# Supplementary material for: Genome-Wide Association Study of d-Amphetamine Response in Healthy Volunteers Identifies Putative Associations, Including Cadherin 13 (CDH13)
Source: PLoS One. 2012 Aug 28;7(8):e42646. doi: 10.1371/journal.pone.0042646 (PMC3429486; doi:10.1371/journal.pone.0042646)
Supplement: Figure S2 — Factor loadings and GWAS results for all 10 factors. Panel A shows the factor loadings; abbreviations of outcome measures are defined in the text. Percentage of variance explained (PVE), or the contribution to the variance in the survey data by each factor, is shown in the top left hand corner. Note that the PVE does not sum, because there is correlation among the factors. Panel B shows the raw phenotype scores as a function of dose (denoted by color coded vertically stacked panels) and time (x-axis within each panel) for individuals in the upper and lower deciles of this factor. Panel C shows the Manhattan plot of observed −log10 P for each chromosome; the red horizontal line indicates 5×10^−8^, which is often used as a threshold for significance. The blue horizontal line indicates 1×10^−5^, which could be considered a threshold for suggestive evidence. Panel D shows a Q-Q plot of observed −log10 P versus the average −log10 P from then random permutations. Factor descriptions are as follows (defined by Panels A and B): F1) responses during the 10 mg and 20 mg sessions, with highest scores on the 10 mg session; F2) positive affect at baseline for all three sessions; F3) responses during the 10 mg and 20 mg sessions, with highest scores on the 20 mg session; F4) responses during the 10 mg and 20 mg sessions; F5) negative affect at baseline for all three sessions; F6) blood pressure baseline measurements for all sessions; F7) responses primarily during the placebo session; F8) baseline measurements for the placebo session; F9) baseline measurements for the 10 mg session; F10) baseline measurements for the 20 mg session. (PDF) [file pone.0042646.s002.pdf]

F1

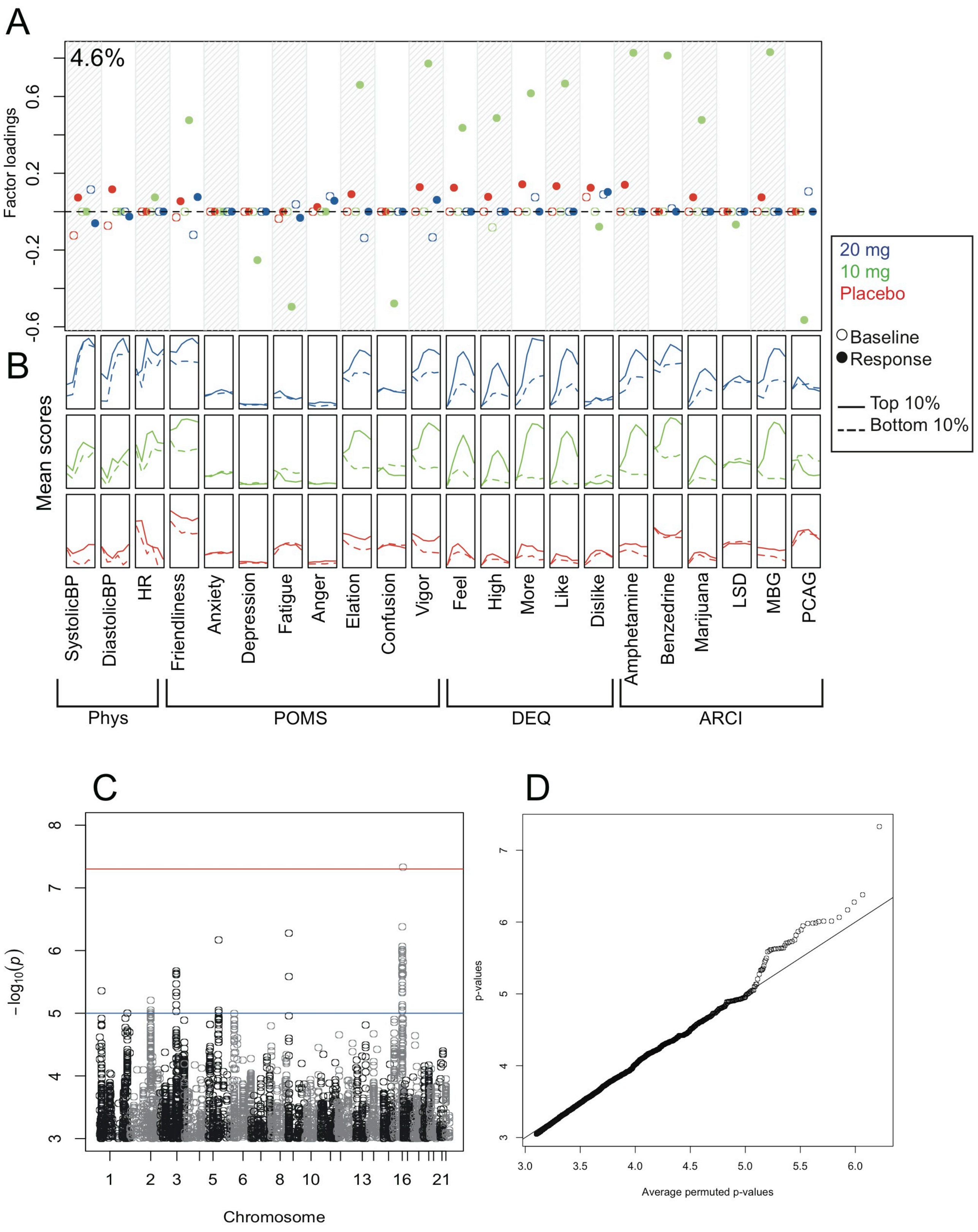

A
4.6%
Factor loadings
B
Mean scores
SystolicBP
DiastolicBP
HR
Friendliness
Anxiety
Depression
Fatigue
Anger
Elation
Confusion
Vigor
Feel
High
More
Like
Dislike
Amphetamine
Benzedrine
Marijuana
LSD
MBG
PCAG
Phys
POMS
DEQ
ARCI
20 mg
10 mg
Placebo
Baseline
Response
Top 10%
Bottom 10%
C
-log10(p)
Chromosome
D
p-values
Average permuted p-values

F2

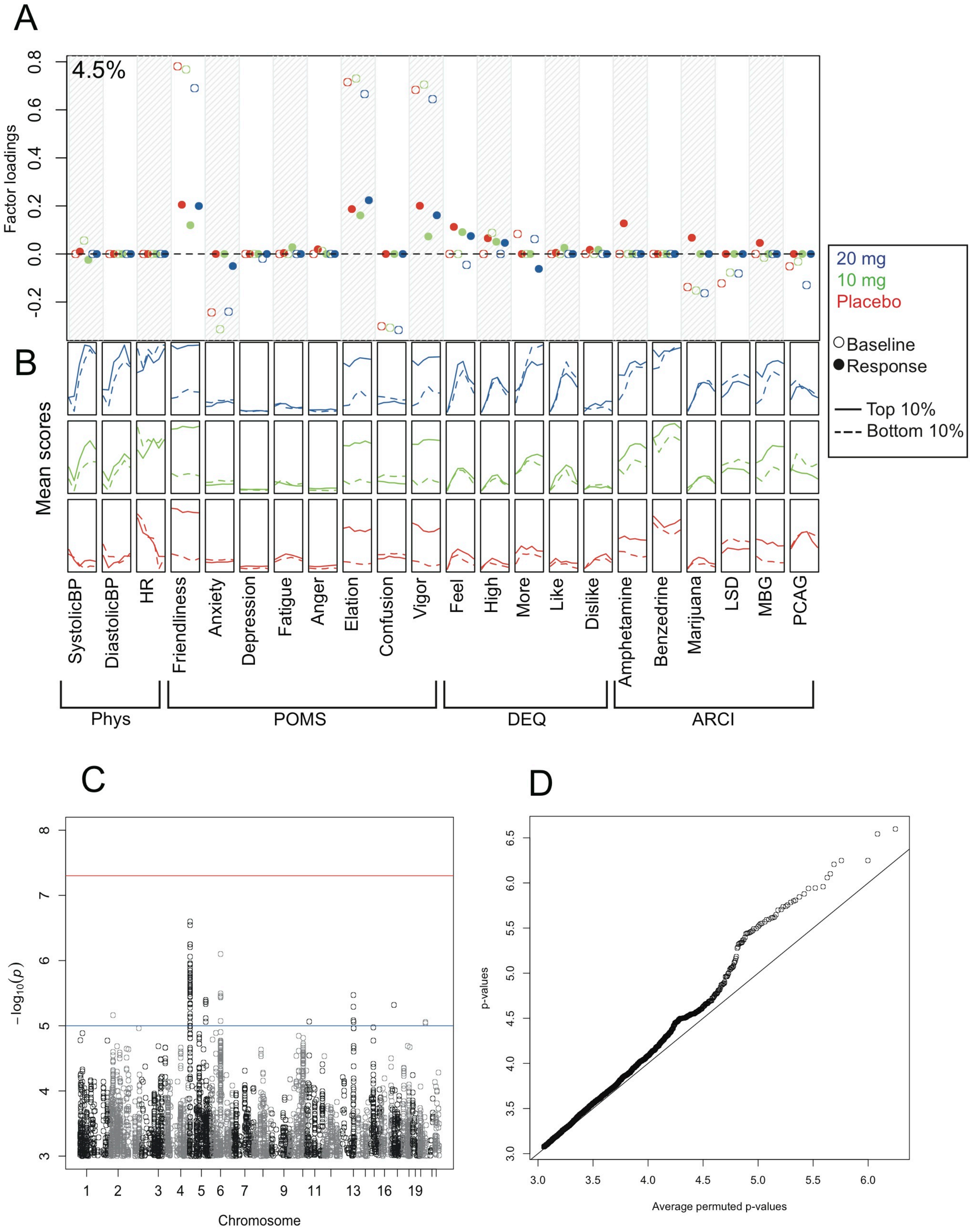

A
4.5%
Factor loadings
20 mg
10 mg
Placebo
Baseline
Response
B
Mean scores
Top 10%
Bottom 10%
SystolicBP
DiastolicBP
HR
Friendliness
Anxiety
Depression
Fatigue
Anger
Elation
Confusion
Vigor
Feel
High
More
Like
Dislike
Amphetamine
Benzedrine
Marijuana
LSD
MBG
PCAG
Phys
POMS
DEQ
ARCI
C
$-\log_{10}(p)$
Chromosome
D
p-values
Average permuted p-values

F3

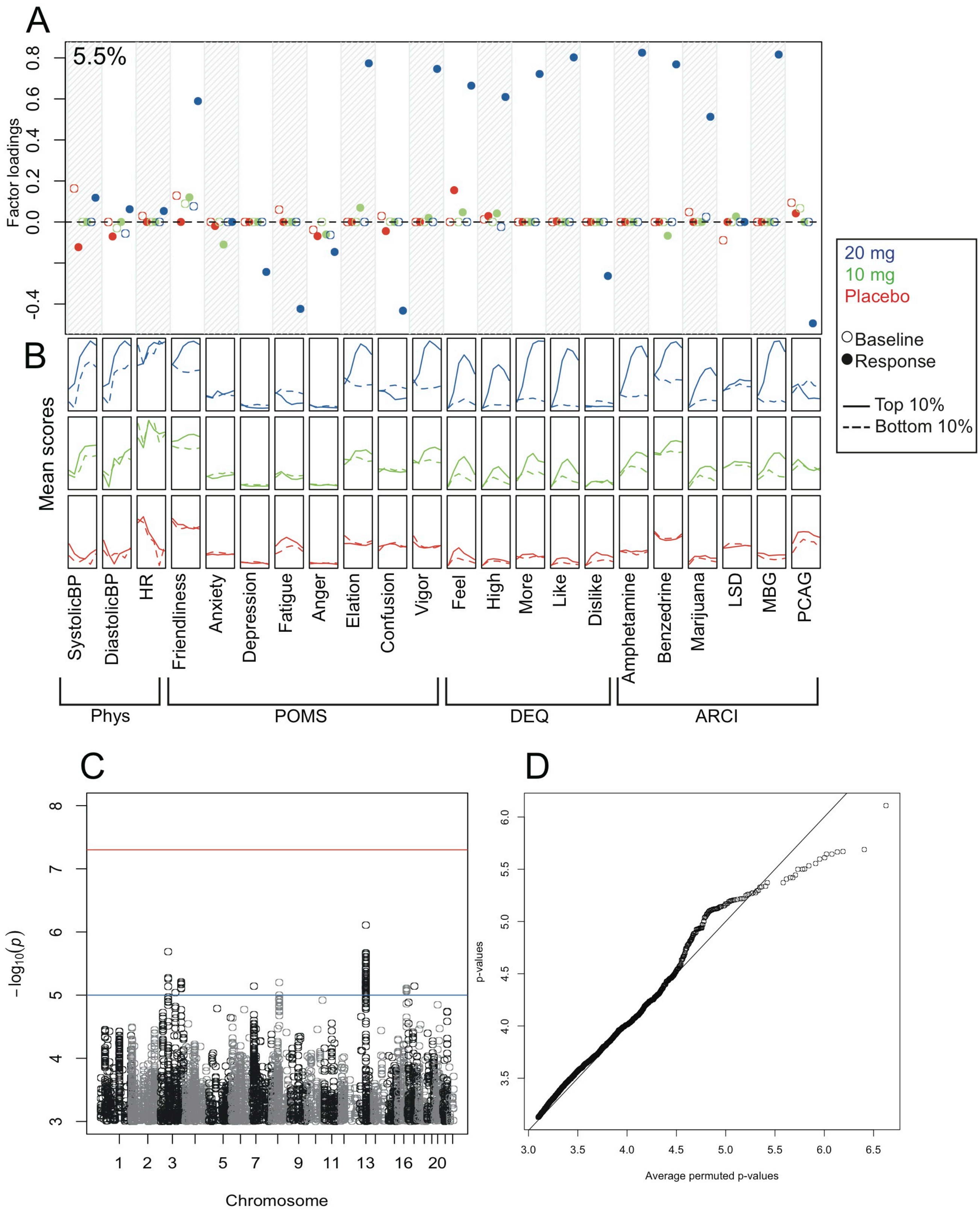
A
5.5%
Factor loadings
0.8
0.6
0.4
0.2
0.0
-0.4
20 mg
10 mg
Placebo
Baseline
Response
Top 10%
Bottom 10%
B
Mean scores
SystolicBP
DiastolicBP
HR
Friendliness
Anxiety
Depression
Fatigue
Anger
Elation
Confusion
Vigor
Feel
High
More
Like
Dislike
Amphetamine
Benzedrine
Marijuana
LSD
MBG
PCAG
Phys
POMS
DEQ
ARCI
C
$-\log_{10}(p)$
Chromosome
1
2
3
5
7
9
11
13
16
20
D
p-values
Average permuted p-values

F4

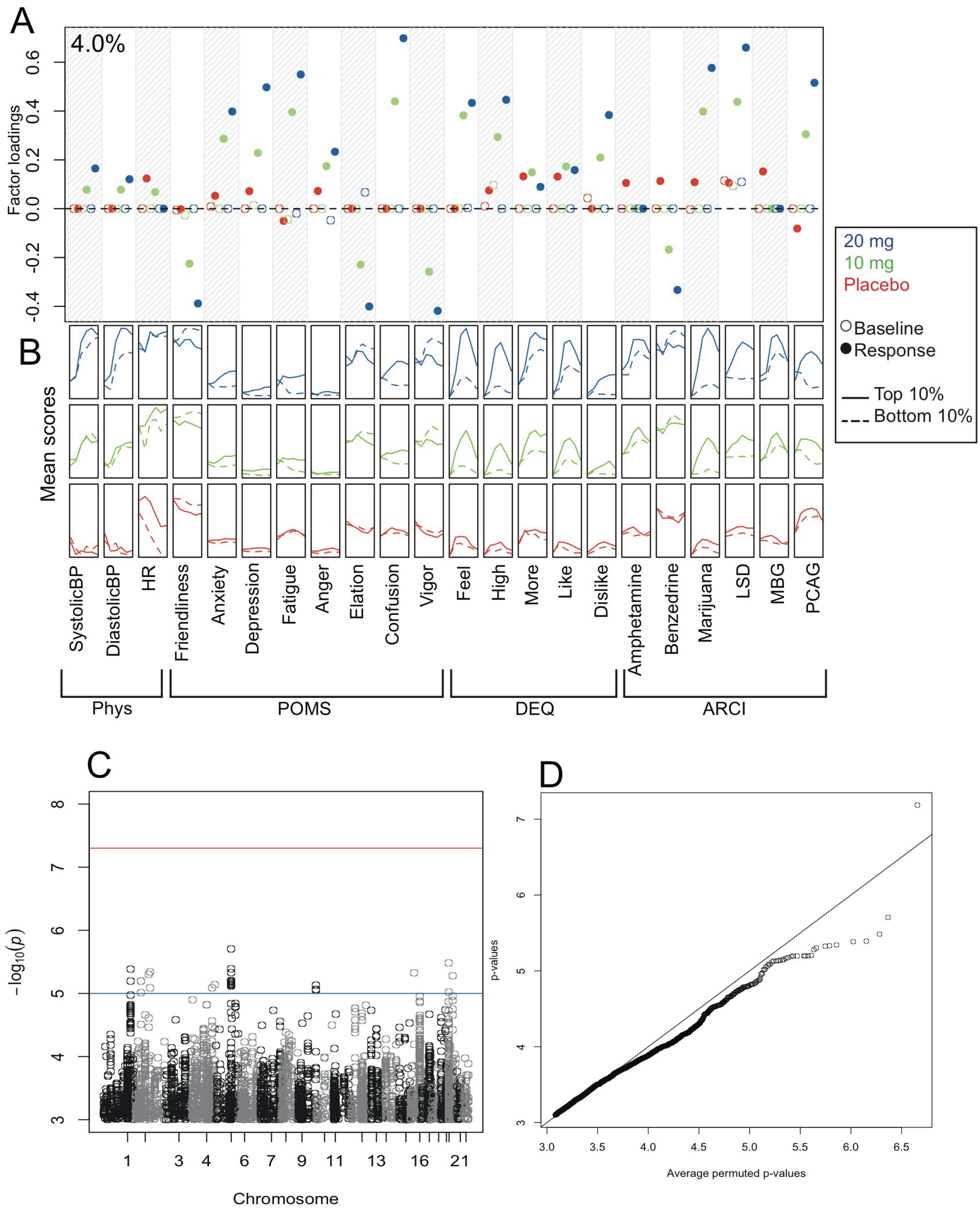
A
4.0%
Factor loadings
0.6
0.4
0.2
0.0
-0.2
-0.4
20 mg
10 mg
Placebo
Baseline
Response
Top 10%
Bottom 10%
B
Mean scores
SystolicBP
DiastolicBP
HR
Friendliness
Anxiety
Depression
Fatigue
Anger
Elation
Confusion
Vigor
Feel
High
More
Like
Dislike
Amphetamine
Benzedrine
Marijuana
LSD
MBG
PCAG
Phys
POMS
DEQ
ARCI
C
-log10(p)
8
7
6
5
4
3
1
3
4
6
7
9
11
13
16
21
Chromosome
D
p-values
7
6
5
4
3
3.0
3.5
4.0
4.5
5.0
5.5
6.0
6.5
Average permuted p-values

F5

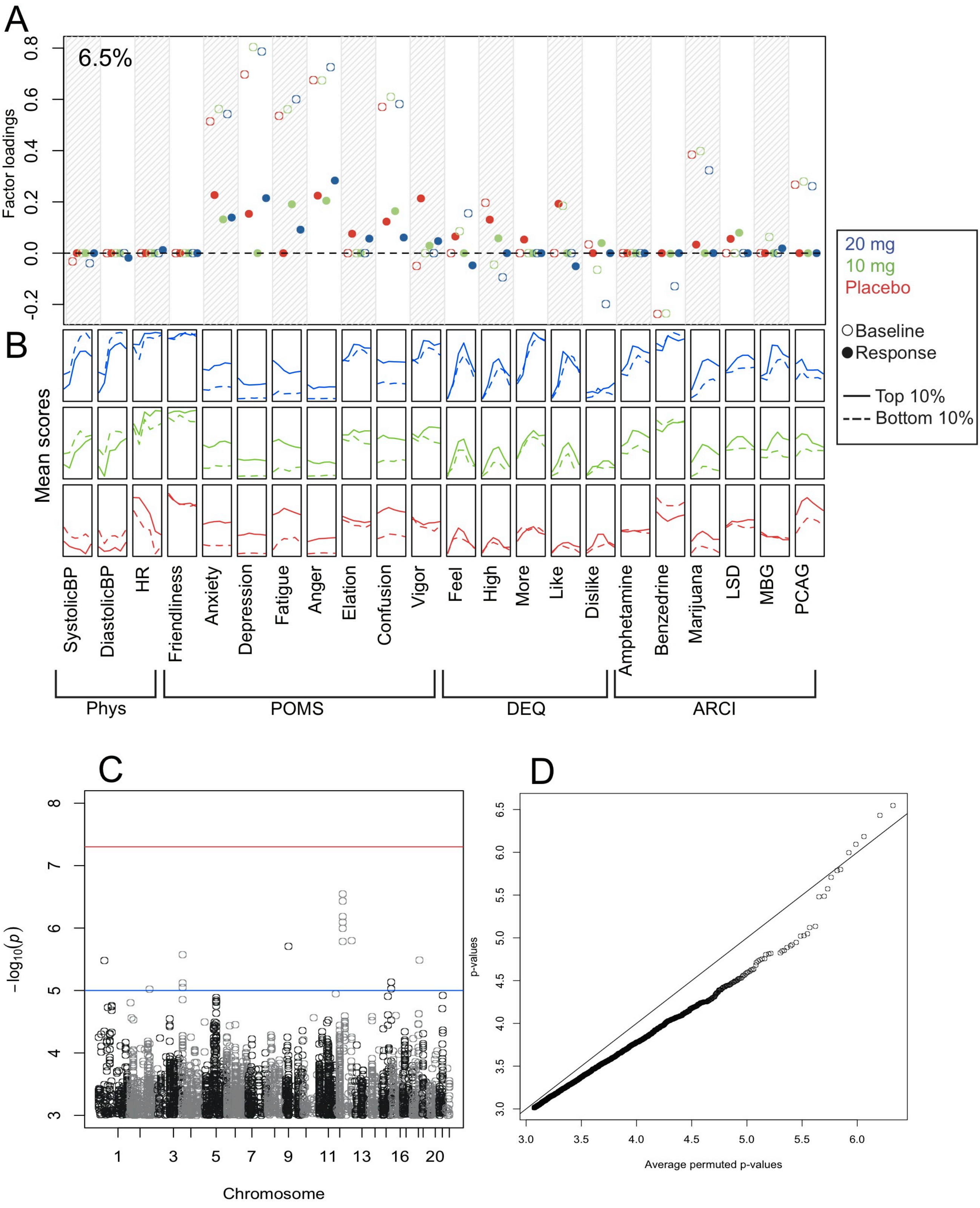
A
6.5%
Factor loadings
20 mg
10 mg
Placebo
Baseline
Response
Top 10%
Bottom 10%
B
Mean scores
SystolicBP
DiastolicBP
HR
Friendliness
Anxiety
Depression
Fatigue
Anger
Elation
Confusion
Vigor
Feel
High
More
Like
Dislike
Amphetamine
Benzedrine
Marijuana
LSD
MBG
PCAG
Phys
POMS
DEQ
ARCI
C
$-\log_{10}(p)$
Chromosome
D
p-values
Average permuted p-values

F6

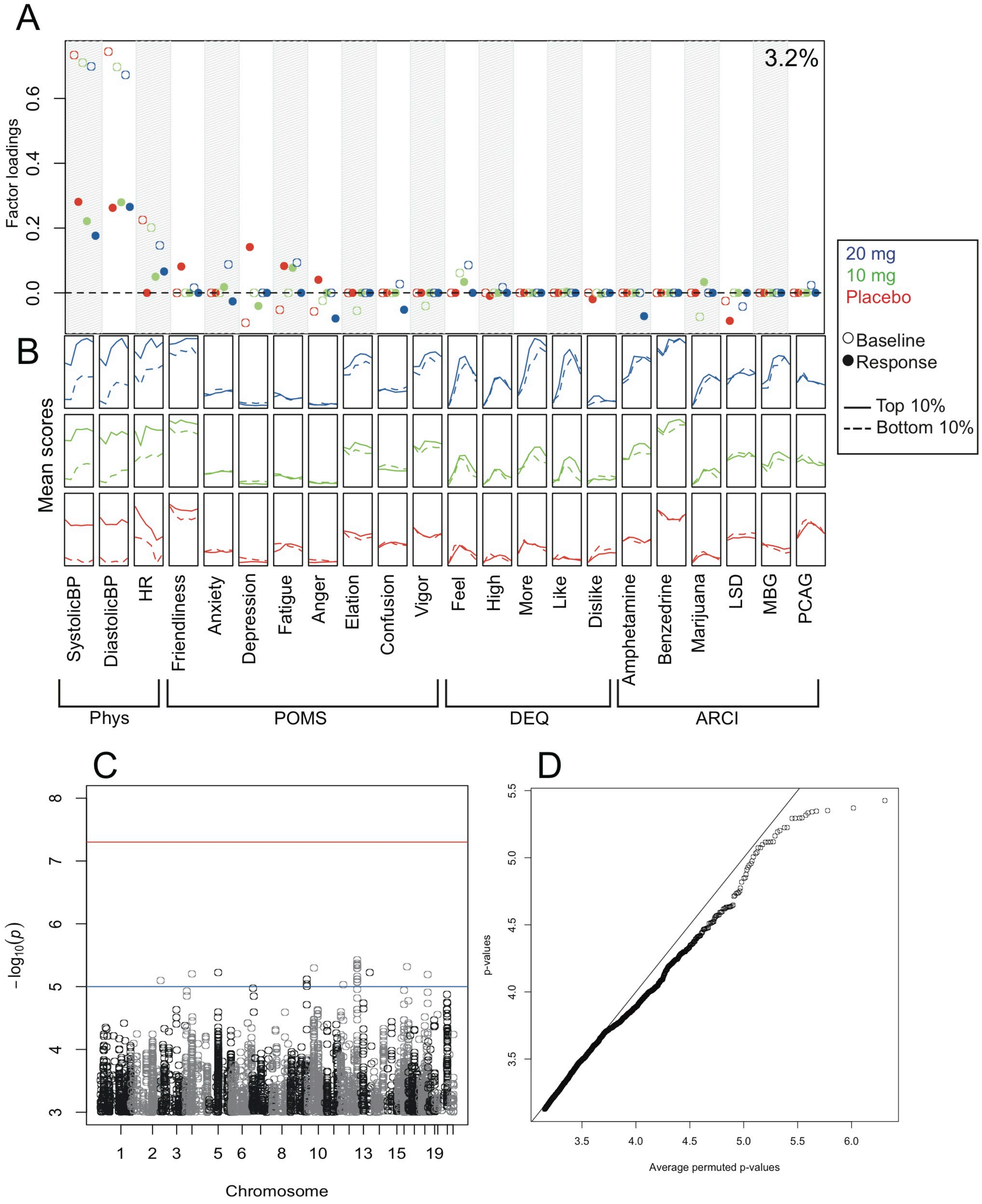
A
3.2%
Factor loadings
20 mg
10 mg
Placebo
Baseline
Response
Top 10%
Bottom 10%
B
Mean scores
SystolicBP
DiastolicBP
HR
Friendliness
Anxiety
Depression
Fatigue
Anger
Elation
Confusion
Vigor
Feel
High
More
Like
Dislike
Amphetamine
Benzedrine
Marijuana
LSD
MBG
PCAG
Phys
POMS
DEQ
ARCI
C
$-\log_{10}(p)$
Chromosome
D
p-values
Average permuted p-values

F7

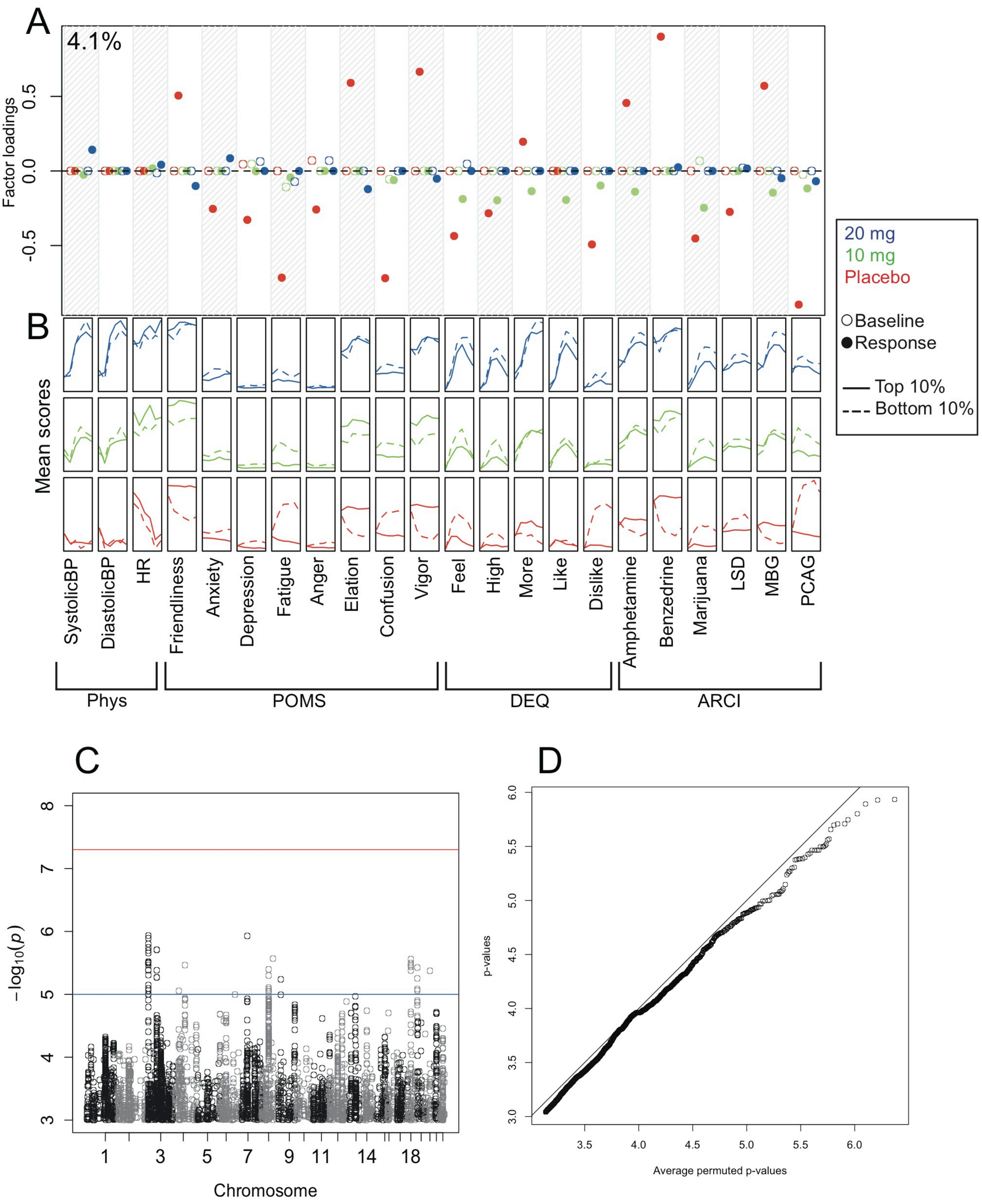

A
4.1%
Factor loadings
0.5
0.0
-0.5
20 mg
10 mg
Placebo
Baseline
Response
Top 10%
Bottom 10%
B
Mean scores
SystolicBP
DiastolicBP
HR
Friendliness
Anxiety
Depression
Fatigue
Anger
Elation
Confusion
Vigor
Feel
High
More
Like
Dislike
Amphetamine
Benzedrine
Marijuana
LSD
MBG
PCAG
Phys
POMS
DEQ
ARCI
C
−log10(p)
8
7
6
5
4
3
1
3
5
7
9
11
14
18
Chromosome
D
p-values
6.0
5.5
5.0
4.5
4.0
3.5
3.0
Average permuted p-values

F8

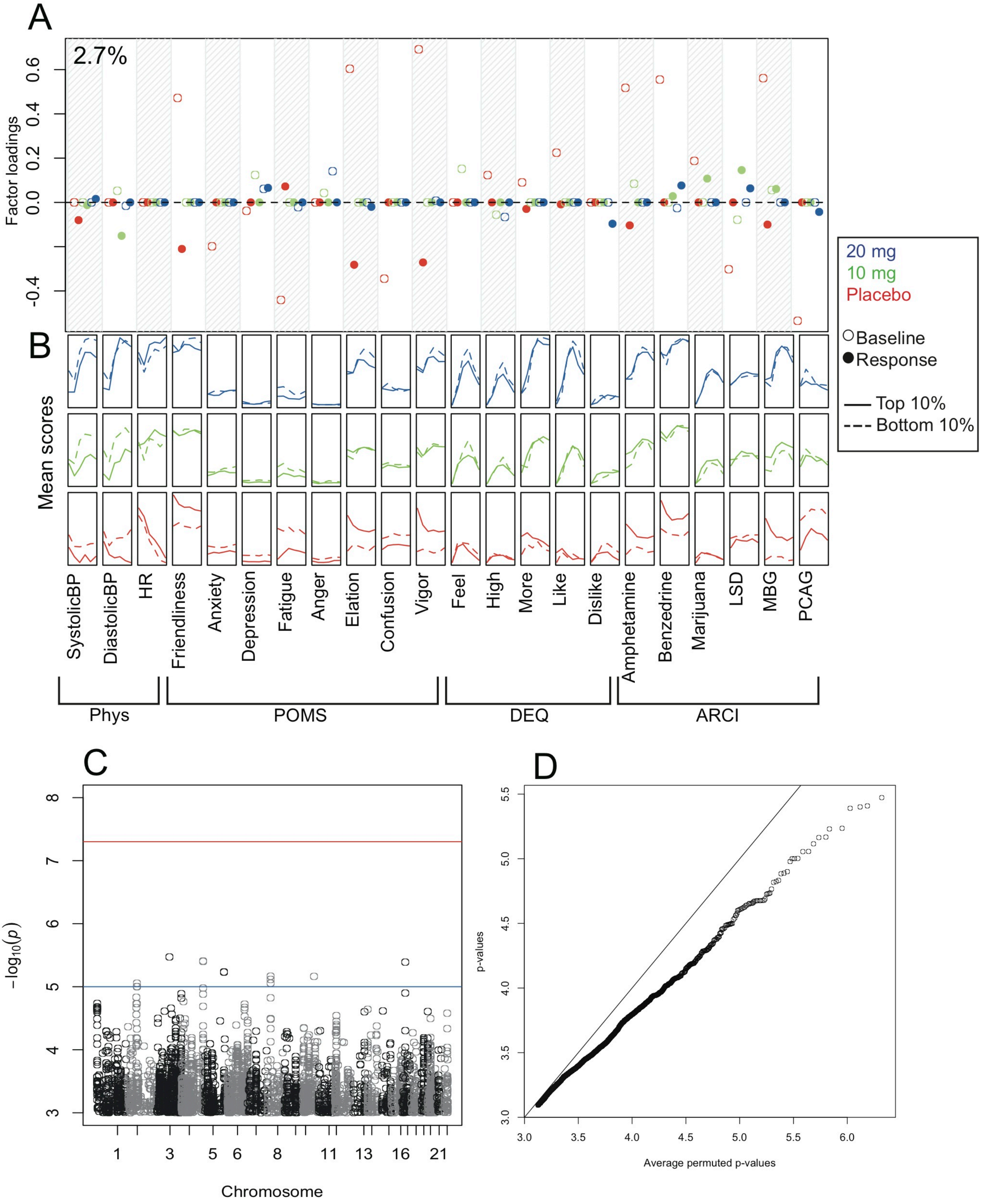
A
2.7%
Factor loadings
0.6
0.4
0.2
0.0
-0.4
20 mg
10 mg
Placebo
Baseline
Response
Top 10%
Bottom 10%
B
Mean scores
SystolicBP
DiastolicBP
HR
Friendliness
Anxiety
Depression
Fatigue
Anger
Elation
Confusion
Vigor
Feel
High
More
Like
Dislike
Amphetamine
Benzedrine
Marijuana
LSD
MBG
PCAG
Phys
POMS
DEQ
ARCI
C
8
7
6
5
4
3
−log10(p)
1
3
5
6
8
11
13
16
21
Chromosome
D
5.5
5.0
4.5
4.0
3.5
3.0
p-values
3.0
3.5
4.0
4.5
5.0
5.5
6.0
Average permuted p-values

F9

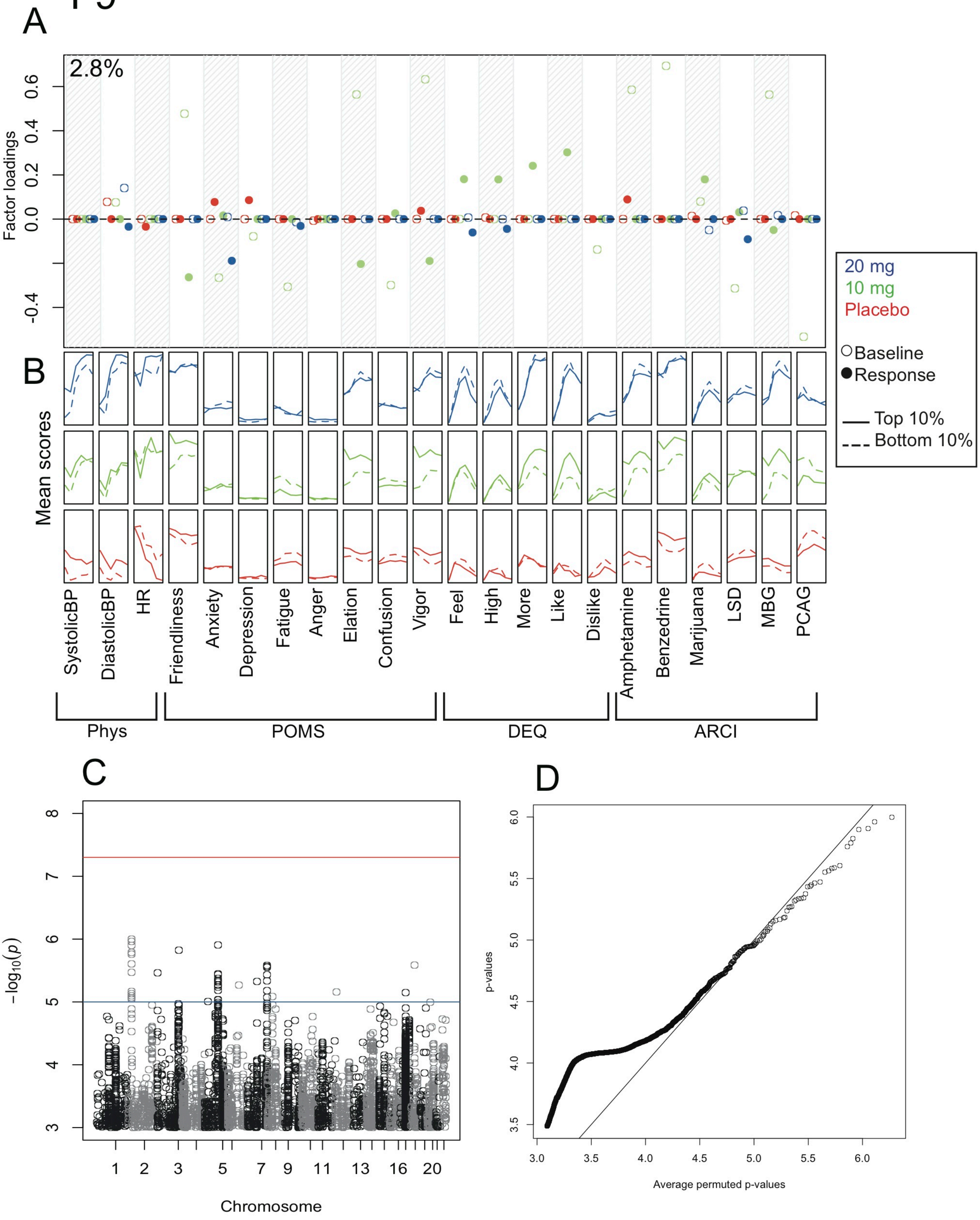
A
2.8%
Factor loadings
0.6
0.4
0.2
0.0
-0.4
20 mg
10 mg
Placebo
Baseline
Response
Top 10%
Bottom 10%
B
Mean scores
SystolicBP
DiastolicBP
HR
Friendliness
Anxiety
Depression
Fatigue
Anger
Elation
Confusion
Vigor
Feel
High
More
Like
Dislike
Amphetamine
Benzedrine
Marijuana
LSD
MBG
PCAG
Phys
POMS
DEQ
ARCI
C
−log10(p)
8
7
6
5
4
3
1
2
3
5
7
9
11
13
16
20
Chromosome
D
p-values
6.0
5.5
5.0
4.5
4.0
3.5
3.0
3.5
4.0
4.5
5.0
5.5
6.0
Average permuted p-values

F10

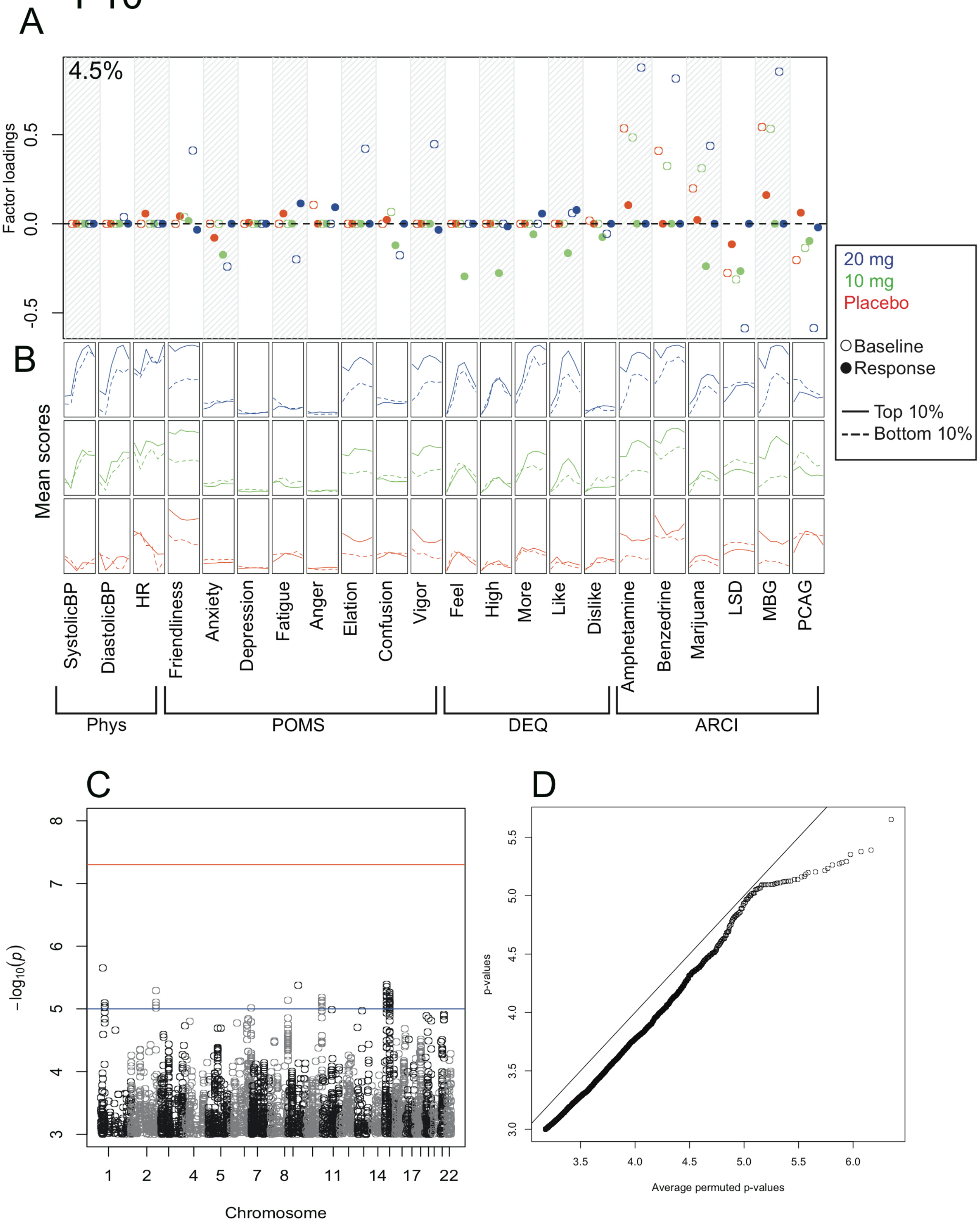

A
4.5%
Factor loadings
0.5
0.0
-0.5
20 mg
10 mg
Placebo
Baseline
Response
Top 10%
Bottom 10%
B
Mean scores
SystolicBP
DiastolicBP
HR
Friendliness
Anxiety
Depression
Fatigue
Anger
Elation
Confusion
Vigor
Feel
High
More
Like
Dislike
Amphetamine
Benzedrine
Marijuana
LSD
MBG
PCAG
Phys
POMS
DEQ
ARCI
C
$-\log_{10}(p)$
3
4
5
6
7
8
1
2
4
5
7
8
11
14
17
22
Chromosome
D
p-values
3.0
3.5
4.0
4.5
5.0
5.5
3.5
4.0
4.5
5.0
5.5
6.0
Average permuted p-values
