# Supplementary material for: Genome-Wide Association Study of d-Amphetamine Response in Healthy Volunteers Identifies Putative Associations, Including Cadherin 13 (CDH13)
Source: PLoS One. 2012 Aug 28;7(8):e42646. doi: 10.1371/journal.pone.0042646 (PMC3429486; doi:10.1371/journal.pone.0042646)
Supplement: Table S1 — Demographic characteristics of the participant sample. Mean values are expressed as mean ± SEM. (DOC) [file pone.0042646.s004.doc]

**Table S1. Demographic characteristics of the participant sample.** Mean values are expressed as mean ± SEM.

| **Demographic Category** | **Demographic** | **Value** |
| --- | --- | --- |
| General | N | 381 |
| General | Age (mean years ± SEM) | 23.2±3.6 |
| General | Gender (% male) | 52 |
| General | Education level – % High school or some college | 43.3 |
| General | Education level – % College degree | 46.5 |
| General | Education level – % Advanced | 10.2 |
| General | BMI (mean + SEM) | 22.6±2.1 |
| Ancestry | % American Indian | 0.2 |
| Ancestry | % African American | 4.9 |
| Ancestry | % Asian | 2.6 |
| Ancestry | % Caucasian | 85.3 |
| Ancestry | % Hispanic | 4.4 |
| Ancestry | % more than one race | 1.6 |
| Ancestry | % missing | 1 |
| Current drug use | Alcohol (mean drinks per week ± SEM) | 5.1±4.3 |
| Current drug use | Cigarettes (mean cigs per week ± SEM) | 0.9±2.1 |
| Current drug use | Caffeine (mean cups per week ± SEM) | 7.8±6.7 |
| Current drug use | Marijuana (mean times per month ± SEM) | 1.4±3.9 |
| Lifetime substance use (ever used) | Sedatives (% yes) | 7.8 |
| Lifetime substance use (ever used) | Stimulants (% yes) | 24.4 |
| Lifetime substance use (ever used) | Opiates (% yes) | 22.5 |
| Lifetime substance use (ever used) | Hallucinogens (% yes) | 34.2 |
| Lifetime substance use (ever used) | Inhalants (% yes) | 10.2 |
| Lifetime substance use (ever used) | Marijuana (% yes) | 75.8 |
