# Supplementary material for: Genome-Wide Association Study of d-Amphetamine Response in Healthy Volunteers Identifies Putative Associations, Including Cadherin 13 (CDH13)
Source: PLoS One. 2012 Aug 28;7(8):e42646. doi: 10.1371/journal.pone.0042646 (PMC3429486; doi:10.1371/journal.pone.0042646)
Supplement: Table S2 — Amphetamine effects on individual scales. POMS is Profile of Mood States questionnaire; DEQ is Drug Effect Questionnaire; ARCI is Addiction Research Center Inventory questionnaire. LSD is Lysergic acid; MBG is Morphine-Benzedrine Group; PCAG is Pentobarbitol-Chlorpromazine-Alcohol Group. (DOC) [file pone.0042646.s005.doc]

**Table S2. Amphetamine effects on individual scales.** POMS is Profile of Mood States questionnaire; DEQ is Drug Effect Questionnaire; ARCI is Addiction Research Center Inventory questionnaire. LSD is Lysergic acid; MBG is Morphine-Benzedrine Group; PCAG is Pentobarbitol-Chlorpromazine-Alcohol Group.

| **Dependent Variable Class** | **Dependent Variable** | **ANOVA: Drug x Time Interactions** | ***P*-value** | **Effect Size (η_p_^2^)** | **Direction of Effect** |
| --- | --- | --- | --- | --- | --- |
| Physiological | Systolic Blood Pressure | F(10, 3800) = 109.53 | 1.42 x 10^-200^ | 0.22 | ↑ |
| Physiological | Diastolic Blood Pressure | F(10, 3800) = 80.87 | 3.22 x 10^-151^ | 0.18 | ↑ |
| Physiological | Heart Rate | F(10, 3800) = 55.44 | 6.17 x 10^-105^ | 0.13 | ↑ |
| POMS | Friendliness | F(10, 3800) = 30.40 | 5.37 x 10^-57^ | 0.07 | ↑ |
| POMS | Anxiety | F(10, 3800) = 6.27 | 1.34 x 10^-9^ | 0.02 | ↑ |
| POMS | Depression | F(10, 3800) = 2.04 | 0.03 | 0.005 | -- |
| POMS | Fatigue | F(10, 3800) = 15.27 | 3.17 x 10^-50^ | 0.07 | ↓ |
| POMS | Anger | F(10, 3800) = 1.58 | 0.11 | 0.004 | -- |
| POMS | Elation | F(10, 3800) = 52.14 | 9.40 x 10^-99^ | 0.12 | ↑ |
| POMS | Confusion | F(10, 3800) = 8.59 | 5.04 x 10^-14^ | 0.02 | ↓ |
| POMS | Vigor | F(10, 3800) = 71.82 | 4.97 x 10^-135^ | 0.16 | ↑ |
| DEQ | Feel | F(10, 3800) = 52.63 | 1.13 x 10^-99^ | 0.12 | ↑ |
| DEQ | High | F(10, 3800) = 45.37 | 6.13 x 10^-86^ | 0.11 | ↑ |
| DEQ | More | F(10, 3800) = 73.81 | 2.25 x 10^-16^ | 0.16 | ↑ |
| DEQ | Like | F(10, 3800) = 67.92 | 5.82 x 10^-128^ | 0.15 | ↑ |
| DEQ | Dislike | F(10, 3800) = 2.90 | 0.001 | 0.008 | -- |
| ARCI | Amphetamine | F(10, 3800) = 72.50 | 3.01 x 10^-136^ | 0.16 | ↑ |
| ARCI | Benzedrine | F(10, 3800) = 49.74 | 3.16 x 10^-94^ | 0.12 | ↑ |
| ARCI | Marijuana | F(10, 3800) = 63.80 | 2.04 x 10^-120^ | 0.14 | ↑ |
| ARCI | LSD | F(10, 3800) = 13.13 | 6.76 x 10^-23^ | 0.03 | ↑ |
| ARCI | MBG | F(10, 3800) = 66.72 | 9.10 x 10^-126^ | 0.15 | ↑ |
| ARCI | PCAG | F(10, 3800) = 24.41 | 3.16 x 10^-45^ | 0.06 | ↓ |
