# Supplementary material for: Genome-Wide Association Study of d-Amphetamine Response in Healthy Volunteers Identifies Putative Associations, Including Cadherin 13 (CDH13)
Source: PLoS One. 2012 Aug 28;7(8):e42646. doi: 10.1371/journal.pone.0042646 (PMC3429486; doi:10.1371/journal.pone.0042646)
Supplement: Table S3 — Description of factors obtained from Sparse Factor Analysis. Interpretations based on factor loading plots (Figure S2A) and decile plots (Figure S2B) are given. PVE refers to percentage of variance explained by each individual factor. (DOC) [file pone.0042646.s006.doc]

**Table S3. Description of factors obtained from Sparse Factor Analysis.** Interpretations based on factor loading plots (Figure S2A) and decile plots (Figure S2B) are given. PVE refers to percentage of variance explained by each individual factor.

| **Factor** | **Name** | **Factor loading plot** | **Decile plot** | **PVE (%)** |
| --- | --- | --- | --- | --- |
| F1 | 10 mg response | This factor captures participants’ response to drug for the 10 mg drug session with substantial positive loadings on positive subjective effect subscales and substantial negative loadings on negative subjective effect subscales. | The decile plot reflects the participants’ scores on the subscales representing drug response in the 10 mg session. Due to correlations between F1 and F3, the 20 mg drug response factor, there is a similar drug response on the 20 mg subscales in the upper and lower deciles of the factor. Additionally, we see little effect on the negative subscales that loaded onto F1, as the majority of the participants have zero scores for these measures during the drug sessions. This upper decile of this factor captures individuals that responded positively to the drug during the 10 mg and 20 mg sessions. | 4.6 |
| F2 | Baseline positive affect | This factor captures participants’ positive affect at baseline, with substantial positive loadings on subscales related to positive affect and substantial negative loadings on subscales related to negative affect. | The decile plot reflects participants’ scores on the subscales describing positive affect across all sessions and consistent across time points. Although negative subscales load onto F2, we see little effect in the decile plot, as the majority of the participants have zero scores for these measures during the drug sessions. The upper decile of this factor captures individuals that have a greater positive affect, independent of drug, time point, and session. | 4.5 |
| F3 | 20 mg response | This factor captures participants’ response to the drug for the 20 mg drug session with substantial positive loadings on positive subjective effect subscales and substantial negative loadings on negative subjective effect subscales. | Due to correlations between F3 and F1, the 10 mg factor, we see minor effects on the 10 mg subscales in the upper and lower deciles of the factor. Additionally, we see little effect on the negative subscales that loaded onto F3, as the majority of the participants have zero scores for these measures. The upper decile of this factor captures individuals that responded positively to the drug during the 10 mg and 20 mg sessions. | 5.5 |
| F4 | 10 and 20 mg response | This factor captures participants’ response to the drug for the 10 mg and 20 mg drug sessions. Substantial positive loadings are on subjective response scales and substantial negative loadings are on negative subjective response subscales. | The decile plot reflects a negative drug response in both the 10 mg and 20 mg sessions. The upper decile of this factor captures individuals that responded to the drug in both the 10 mg and 20 mg session, but did not like the way the response felt. | 4.0 |
| F5 | Baseline negative affect | This factor captures participants’ negative affect at baseline, with substantial positive loadings on subscales representing negative affect across all sessions. | The decile plot reflects participants’ scores on the subscales describing negative affect across all sessions and consistent across time points. The upper decile of this factor captures individuals that have a greater negative affect, independent of drug, time point, and session. | 6.5 |
| F6 | Baseline blood pressure | This factor captures participants’ blood pressure readings at baseline across all sessions. | The decile plot reflects participants’ blood pressure readings across all sessions and all time points, as well as reveals an effect on heart rate, which is correlated with blood pressure. The upper decile of this factor captures individuals who have higher blood pressure across all sessions and time points. | 3.2 |
| F7 | Placebo response | This factor captures participants’ response to placebo, with substantial positive loadings on positive subjective effect subscales and substantial negative loadings on negative subjective effect subscales. | The decile plot shows individuals in the upper decile have the highest mean scores on positive subjective subscales and lowest mean scores on the negative subjective effect scales. The lower decile of this factor captures individuals who show a sedative drug effect during the placebo session. | 4.1 |
| F8 | Placebo baseline | This factor captures participants’ baseline subjective ratings for the placebo session, with substantial positive loadings on positive subjective effect subscales and substantial negative loadings on negative effect subscales. | The decile plot closely reflects what is seen in the factor plot: individuals in the upper decile have the highest mean scores on positive subjective subscales and lowest mean scores on the negative subjective effect scales. The upper decile of this factor captures individuals that show less fatigue, higher positive affect, and higher scores on the stimulant ARCI subscales across all time points of the placebo session. | 2.7 |
| F9 | 10 mg baseline | This factor captures participants’ baseline subjective ratings for the 10 mg session, with substantial positive loadings on the positive subjective effect subscales. Minimal loadings are seen for the negative subjective effect scales. | The decile plot reflects subjective response ratings for the 10 mg session across all time points. The upper decile of this factor captures individuals who showed a consistently positive subjective drug effect only during the 10 mg session. | 2.8 |
| F10 | 20 mg baseline | This factor captures participants’ baseline subjective ratings for the 20 mg session, with substantial positive loadings on the positive subjective scales, with the highest loadings on the ARCI subscales. | The decile plot reflects effects on the positive subjective effect subscales, which are consistent across all time points. The upper decile of this factor captures individuals that show both a positive subjective affect across all sessions and time points, with a substantial response on the ARCI subscales, and a positive drug response that is dose dependent. | 4.5 |
