# Supplementary material for: Genome-Wide Association Study of d-Amphetamine Response in Healthy Volunteers Identifies Putative Associations, Including Cadherin 13 (CDH13)
Source: PLoS One. 2012 Aug 28;7(8):e42646. doi: 10.1371/journal.pone.0042646 (PMC3429486; doi:10.1371/journal.pone.0042646)
Supplement: Supporting Information S1 — Combined Supporting Materials and Methods and Supporting Results file. Additional details of the methods are provided in the Supporting Materials and Methods section. Alternative dimensionality methods are detailed in the Supporting Materials and Methods and Results sections. (DOC) [file pone.0042646.s009.doc]

**Supporting Information S1**

Genome-wide association study of *d*-amphetamine response in healthy volunteers identifies putative associations, including cadherin 13 (*CDH13*)

Amy B. Hart^1,5^, Barbara E. Engelhardt^1,2,5^, Margaret C. Wardle^3^, Greta Sokoloff^1^, Matthew Stephens^1,4^, Harriet de Wit^3^, Abraham A. Palmer^1,3*^

^1^Department of Human Genetics, University of Chicago, Chicago, IL

^2^Department of Computer Science, University of Chicago, Chicago, IL

^3^Department of Psychiatry and Behavioral Neuroscience, University of Chicago, Chicago, IL

^4^Department of Statistics, University of Chicago, Chicago, IL

^5^These authors contributed equally

Table of Contents

**Supporting Materials and Methods** [**2**](#__RefHeading___Toc182376028)

Phenotype procedure (additional details) [2](#__RefHeading___Toc182376029)

ANOVA analysis [3](#__RefHeading___Toc182376030)

Alternative phenotypic data summarizations [3](#__RefHeading___Toc182376031)

Caucasian-only sample (additional details) [5](#__RefHeading___Toc182376032)

Genotyping and quality control (additional details) [6](#__RefHeading___Toc182376033)

Supporting Results [7](#__RefHeading___Toc182376034)

Correlation among factor phenotypes [7](#__RefHeading___Toc182376035)

Alternative methods for dimensionality reduction [9](#__RefHeading___Toc182376036)

References [14](#__RefHeading___Toc182376037)

Supporting Methods

In this supporting information, we present more details about the methods used in our study. We also present three alternative phenotype summarization methods and two alternative dimensionality reduction methods (PCA and factor analysis with a varimax rotation).

# Supporting Materials and Methods

## Phenotype procedure (additional details)

The sample consisted of 398 healthy volunteers aged 18 to 35 years (17 participants could not be included in the final analysis as discussed in the latter parts of this supplement). Participants first attended an orientation during which they were familiarized with the questionnaires and procedures. For all sessions, participants arrived at 9:00am and completed breath and urine tests. They then consumed a standard snack and completed the physiological measures and questionnaires for subjective effects (0 minutes). At 9:30am participants were administered two opaque size 00 gelatin capsules containing 10 mg or 20 mg of *d*-amphetamine with dextrose filler, or placebo (dextrose only). They completed additional questionnaires at 10:00am (30 minutes), 10:30am (60 minutes) and 11:00 am (90 minutes), 12:00pm (150 minutes) and 12:30pm (180 minutes). At 11:30 they completed cognitive tasks not reported here. Cardiovascular responses were collected twice at each time point to ensure data quality and these were averaged. The subjective response questionnaires were collected by computer and computer-scored. Participants were allowed to watch a movie or read during sessions when no activities were scheduled. This study was approved by the Institutional Review Board of The University of Chicago and was carried out in accordance with the Helsinki Declaration of 1975.

Subjective responses were measured using three standard questionnaires. The Profile of Mood States (POMS) [1] consists of 72 adjectives rated on 5-point Likert scales from 0 (“not at all”) to 4 (“extremely”), which are linearly combined into eight subscales: Friendliness, Anxiety, Depression, Fatigue, Anger, Elation, Confusion and Vigor. The POMS has previously been shown to be sensitive to the euphoric effects of amphetamine [2]. The Drug Effects Questionnaire (DEQ) [3] consists of a set of visual analog scales on which participants mark from 1 to 100 how much they feel the drug’s effects, feel high, want more of the drug, like the drug’s effects, and dislike the drug’s effects. The Addiction Research Center Inventory (ARCI) [4,5] is an empirically derived 52-item true/false scale with six sub-scales derived from subsets of the 52-item scales that measure the prototypical effects of six classes of drugs: Amphetamine, Benzedrine, Marijuana, Lysergic Acid (LSD), Morphine-Benzedrine Group (MBG) and the Pentobarbitol-Chlorpromazine-Alcohol Group (PCAG).

## ANOVA analysis

To examine effects of amphetamine, we conducted three (dose) by six (time point) repeated-measures ANOVA on the cardiovascular measures, POMS, ARCI and DEQ sub-scales, with partial eta-squared effect sizes using SPSS software version 17.0. The dose by time interactions, which capture effects of amphetamine over the course of each session, are presented in Table S4. We used a Bonferroni-corrected threshold of *P*<2.27x10^-3^ to determine significance.

## Alternative phenotypic data summarizations

We summarized the data in four ways, each of which yielded similar factors. The Methods section in the manuscript describes the summarization method for the results described therein; we call that approach, which did not include the 30 minute time point, ‘n30’ in the following discussion. We also summarized factors in three alternate ways.

030: First, we included time point 30 in the baseline measure by taking the mean of time points 0 and 30 (*baseline*), and (as in the paper) taking the mean of time points 60, 90, 150, and 180 (*response*), and controlling for the baseline measure in the response measure; we call this approach ‘030’. The factors using this summarization method were well correlated with the summarization method used in the paper; however, since time point 30 was correlated with response, controlling for the baseline measure removed some of the response signal from the response measure subscales.

Reverse: Second, we performed the same averaging as in the first alternate summarization method (baseline measure is the average of time points 0 and 30; response measure is the average of time points 60, 90, 150, 180) but controlled the baseline measure subscales for the response measure subscales; we call this approach ‘reverse’. This summarization method also produced factors that were well correlated with the n30 summarization method; however the response measure subscales were not as interpretable because they were confounded with baseline subscales. For example, an individual feeling relatively elated on at time 0 tends to remain more elated throughout the session, and this *session effect* is not as well controlled in this summarization scheme.

Add sub: Third, we created uncorrelated baseline and response subscales by performing the same averaging as the ‘030’ summarization method (baseline measure is the average of time points 0 and 30; response measure is the average of time points 60, 90, 150, 180), and then created the baseline measure subscales by adding the two averaged subscales and the response measure subscales by subtracting the later averaged subscales from the earlier averaged subscales; we call this approach ‘addsub’. This alternative summarization method corresponded less well to the factors produced by the ‘n30’ summarization method, and the interpretability of these factors was lower: while the response measure subscale (i.e., the subtracted averages) is straightforward to interpret as the response minus the baseline, the baseline measure subscale (i.e., the sum of the averages) is less straightforward to interpret in terms of a phenotype. The phenotype should represent the average subscale measurement independent of drug, although since it includes the response measure subscale measurements that have a large variance, those often skew the average.

With these summarized data matrices, we normalized each column, which is either the baseline or the response measure for one question for a single individual at one session, to the standard quantiles, or *quantile normalized* them. As in the manuscript, we ran sparse factor analysis (SFA) [6] with eleven factors and a mean term on the rows. We chose eleven factors by examining the set of factors that were produced by different numbers of factors and selecting the number of factors above which current factors were split instead of new factors being added. Since the initialization is stochastic for SFA, we started each SFA at one hundred random starting points and found 100 different sets of factors. We computed correlation between each set of factors by computing the Pearson correlation between each pair of corresponding factors after 'sorting' the factors in each set to ensure the corresponding factors captured the equivalent phenotype. The run with the highest average correlation with the other 99 factor sets was chosen as most representative. The chosen factor matrix was an 11 × 381 matrix; for each individual, we extracted eleven phenotype values by quantile normalizing the factor matrix. We removed one of the factors because of its high correlation with the known covariate ‘Age’ and were left with ten factors. The set of ten factors is found in Figure S2.

## Caucasian-only sample (additional details)

From each of these sets of factors, we controlled for ﬁrst two genetic PCs from SmartPCA for the 381 individual genotypes, which represent ancestry. We computed the residuals of this model for each of the factors and quantile normalized these residuals by factor; these were our phenotypes for the 381 participants. From these factors, we extracted the self-identified Caucasians that also clustered with the Caucasians in the principal components analysis. We identified two participants that self-identified as Caucasian but clustered with the Hispanic participants in the PCA analysis (Figure S3B); these two participants were not included in the Caucasian-only sample. After extracting the factors for the Caucasian participants, we quantile normalized each phenotype but did not control for structure within the Caucasians; this produced Caucasian-only phenotypes with 325 participants.

## Genotyping and quality control (additional details)

DNA was extracted from blood at the General Clinical Research Center at the University of Chicago with the Qiagen Flexigene DNA AGF3000 Kit (Qiagen; Valencia, CA, USA). In the few cases that blood was not available, DNA was extracted from saliva samples with the Oragene OG-250 or OG-500 kit (Oragene, DNA Genotek; Kanata, Ontario, Canada). DNA samples from eleven participants did meet the required yield to perform genotyping, samples from two participants failed array QC and were not run, and one individual was excluded for a gender discrepancy between phenotype and genotype. Additionally, one individual was excluded for having a missing DNA sample. The second set of phenotyping sessions were removed for two participants who had completed the study twice over a period of approximately two years. These repeat participants’ phenotype responses were well correlated (Pearson correlation of 0.979 and 0.982). We used the earlier study phenotypes for both participants to avoid possible order biasing.

Genotypes were called with the Birdseed [7] and CRLMM [8] algorithms. Genotypes that did not agree between these two methods were set to missing and imputed. SNPs that were mismatched in four or more participants were set to missing in all participants and imputed. Hardy-Weinberg equilibrium (HWE) p-values were computed with PLINK [9] in the Caucasian participants. A Q-Q plot was generated and the cutoff was set at 10^-4^ (Figure S3A). As a result, 149 SNPs were removed and imputed. Heterozygosity and allele sharing were computed with PLINK. We verified that each individual’s self-reported sex and ancestry information matched with our assessment based on genotyping; ancestry was assessed using the SmartPCA component of EIGENSOFT [10] (Figure S3B). Following quality control, 381 participants remained (male=200, female=181). We imputed all remaining genotypes using impute2 software package [11], using the phased HapMap 3 genotypes [12] and 1000 Genomes genotypes [13], available on the Impute2 website, as reference panels. We used an effective population size of 20,000 because of the heterogeneous ancestry of our samples. Imputation brought the total number of SNPs to 7,573,542 for each individual. We removed SNPs that had a MAF < 0.05 in the full sample and were left with 5,476,100 SNPs.

# Supporting Results

## Correlation among factor phenotypes

We can examine a single factor that indicates response to amphetamine more closely, and compare it to similar response factors. The factor loading plot for a response factor with substantial loadings in the 10 mg session questions shows that the questions with larger absolute values for a specific factor contribute to the factor more than those questions with factor loadings near zero (F1; Figure 1A). For this factor, the placebo session and the 20 mg session do not appear to contribute substantially to the factor. In particular, the subscales that are correlated and indicate a positive response to the drug (e.g., Elation and Like) have positive weights, whereas subscales that are correlated and indicate a negative response (e.g., Fatigue, PCAG) have negative weights (the direction and scale of the factor loading is arbitrary).

For comparison, there is an equivalent response factor to the 10 mg session response factor with loadings mostly from the 20 mg session subscales (F3; Supporting Figure A). This factor was correlated with the factor from the 10 mg session (Pearson correlation=0.35). The factor loading plots from the two factors look almost identical, although they represent responses in different sessions.

*
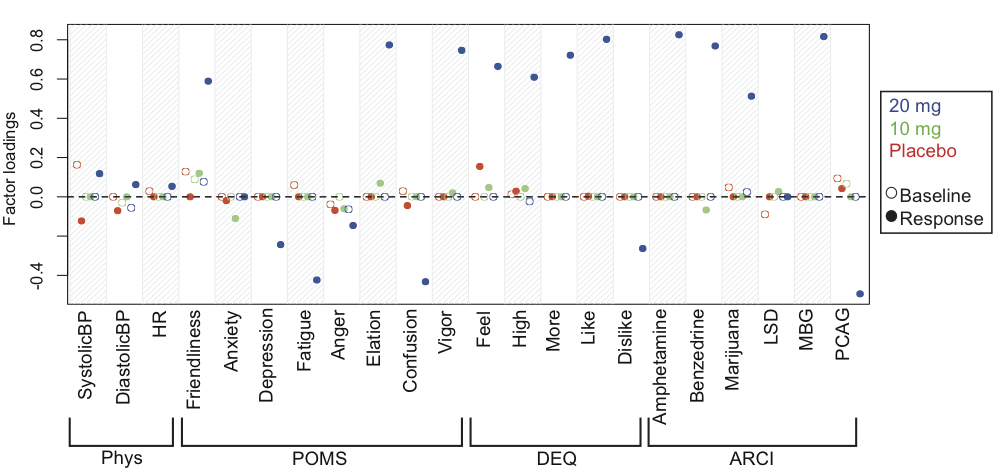
* **Supporting Figure A: Factor loadings for F3.** Factor loadings are plotted for F3, where red indicates placebo session, green indicates 10 mg session and blue indicates 20 mg session; the closed shapes represent the response summary (post-capsule ingestion) and the open shapes represent the baseline summary (pre-capsule ingestion).

The mean top 10% and bottom 10% plots show that the two factors have substantial overlap in their phenotypes (Figure 2E, Supporting Figure B); in particular F1 has substantial increase from placebo in drug-responsive subscales in the 20 mg session, and similarly F3 has substantial increase from placebo in drug response subscales in the 10 mg session. Although these two factors are well correlated, the same genotype (rs3784943) that has a low p-value (p-value = 4.58 x 10^-8^) with F1 has a much higher p-value (p-value = 0.049) with F3.


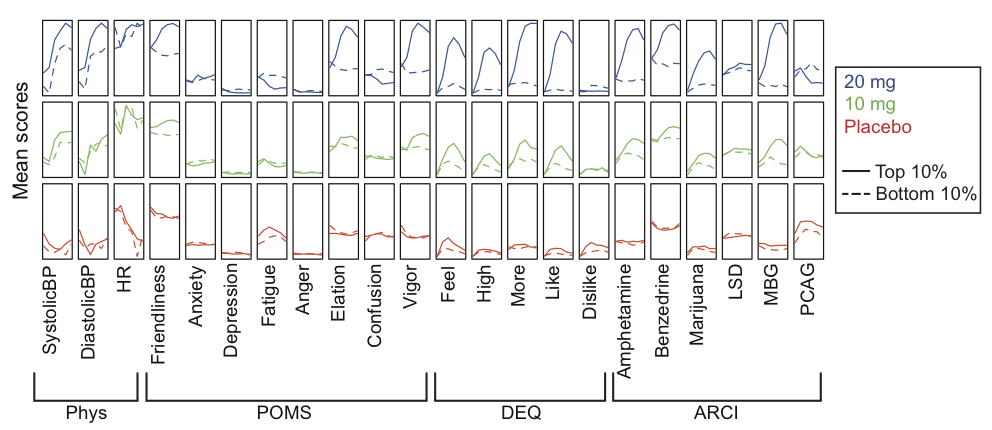


**Supporting Figure B: Mean scores of the top 10% and bottom 10% of individuals for F3.** The 38 individuals with the highest factor values have higher response measure mean values during the 20 mg session (and less substantial increases for the 10 mg session) for the physiological subscales, the positive POMS subscales, the positive DEQ subscales, and stimulant ARCI subscales.

## Alternative methods for dimensionality reduction

In the main text we described how SFA leads to interpretable factors, and here illustrate this interpretability as compared to principal components analysis (PCA) and factor analysis (FA) with varimax rotation. PCA is a method to identify the directions of largest variance in a sample, and each principal component (PC) is orthogonal to the others. The principal components are more difficult to interpret as phenotypes, as they have little sparsity. Factor analysis is a similar model to our SFA, but it does not encourage sparsity in the loadings explicitly; the varimax rotation finds the rotation of the loadings matrix with maximal sparsity by maximizing the sum of the variances of each of the squared factor loadings [14].

The factors produced by SFA have similar representations to the factors produced by FA, but with a much larger proportion of subscales with near-zero loadings (that effectively contribute nothing to the factor). Because PCA requires factors to be uncorrelated, whereas neither FA nor SFA has the same requirement, the individual factors from PCA are often made up of multiple, correlated factors from the FA methods. The first PC (Supporting Figure C) appears to a combination of the baseline positive affect factor (Figure 2A) and baseline negative affect factor (Supporting Figure D), for example.


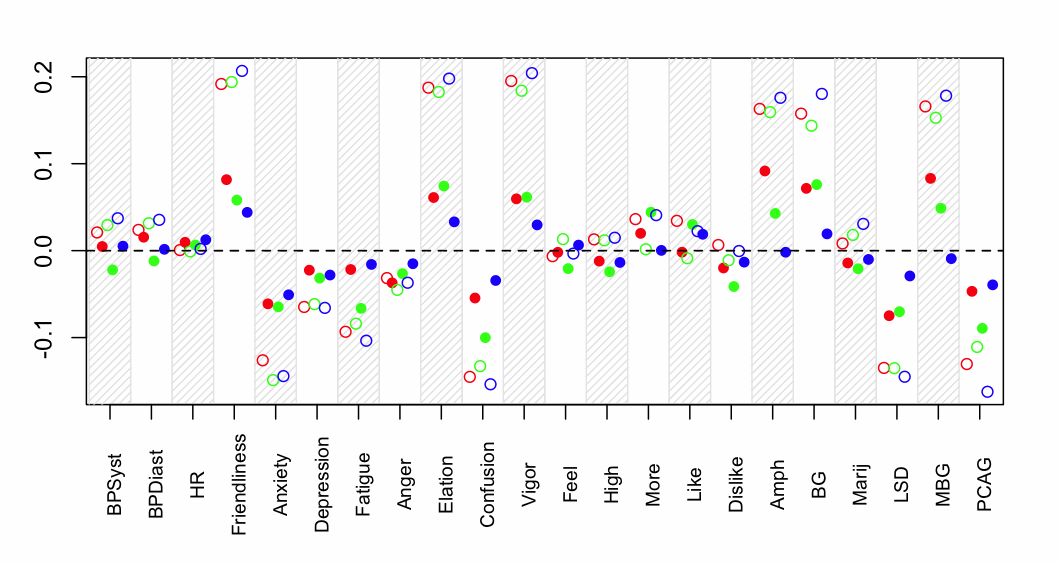


**Supporting Figure C: First principal component from summarized data.** Red indicates placebo session, green indicates 10 mg session and blue indicates 20 mg session; the closed shapes represent the response summary and the open shapes represent the baseline summary.


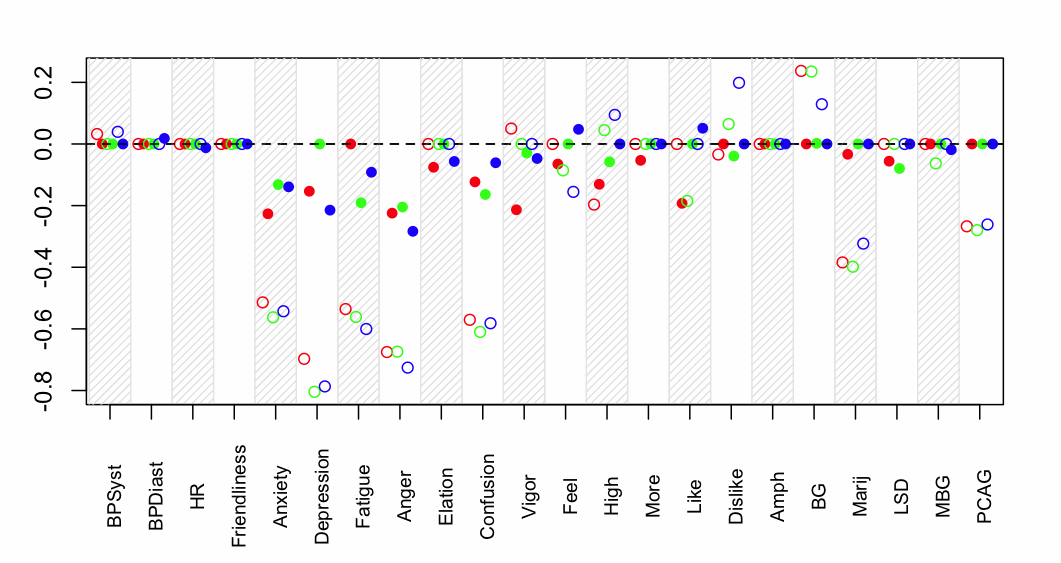


**Supporting Figure D: Factor capturing negative affect at baseline in summarized data.** Red indicates placebo session, green indicates 10 mg session and blue indicates 20 mg session; the filled symbols shapes represent the response measure summary and the open symbols represent the baseline measure summary.

Similarly, the second PC appears to capture a combination of the 10 mg and 20 mg response factor and the placebo response factor, along with having substantial loadings on both baseline and response subscales across the three sessions (Supporting Figure E).


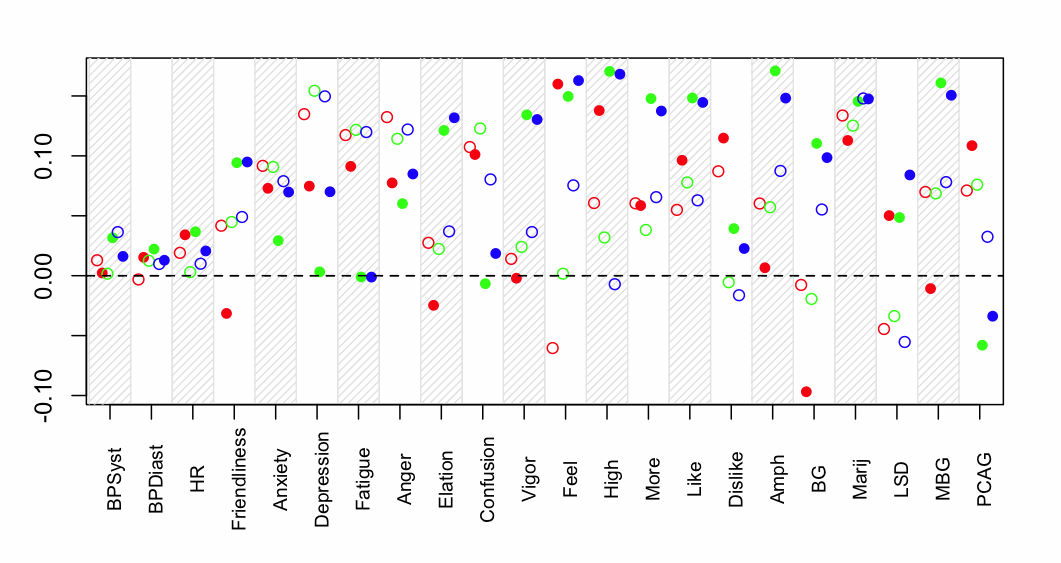


**Supporting Figure E: Second principal component from summarized data.** Red indicates placebo session, green indicates 10 mg session and blue indicates 20 mg session; the closed shapes represent the response summary and the open shapes represent the baseline summary.

By the sixth principal component (Supporting Figure F), the variance captured appears to be a statistical artifact rather than interpretable signal, which is a known product of PCA [15]. It is not clear what phenotype is represented by the sixth PC; the PC appears to separate placebo and 20 mg response session responses from the 10 mg session response. Ultimately, we chose to use SFA over FA with varimax rotation or PCA because of the interpretability of SFA results over the other two methods.


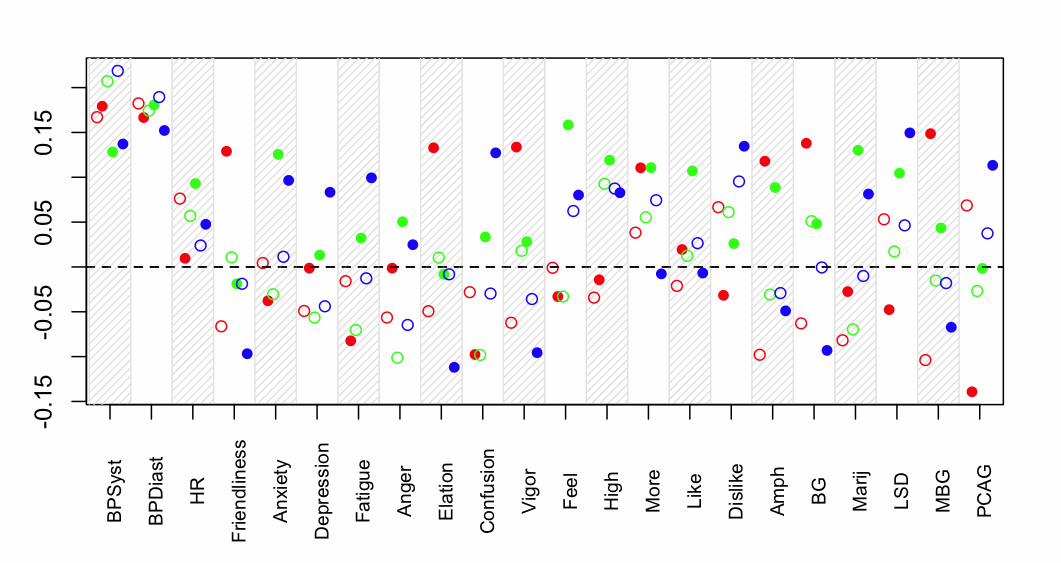


**Supporting Figure F: Sixth principal component from summarized data.** Red indicates placebo session, green indicates 10 mg session and blue indicates 20 mg session; the closed shapes represent the response summary and the open shapes represent the baseline summary.

In this study, we focused on building a high-quality phenotype characterization because of the small sample size and the extensive rich, but noisy, phenotype data collected. There is an implicit trade-off between sample size and the quality of complex traits that are difficult to capture at low cost, both of which contribute to the power to detect associations in a genome-wide association study.

While techniques that aggregate information in an exploratory or confirmatory dimension reduction are attractive, the results must be both interpretable (to ensure a successful mapping will enable explanation of a particular trait) and biologically meaningful (so that the phenotype is a good proxy for the actual trait manipulated by the identified SNP). We can evaluate a specific low-dimensional phenotype mapping’s interpretability. Interpretability is facilitated through sparsity (i.e., only a handful of subscales with substantial loadings for a particular factor) so we can get a clear picture of the phenotype represented by the factor. We have encouraged factor interpretability by putting a sparse prior on the factor loadings in our projection model.

# References

1. Johanson C, Uhlenhuth E (1980) Drug preference and mood in humans: diazepam. Psychopharmacology (Berl) 71: 269-273.

2. Gabbay FH (2003) Variations in affect following amphetamine and placebo: markers of stimulant drug preference. Experimental and Clinical Psychopharmacology 11: 91-101.

3. Fischman MW, Foltin RW (1991) Utility of subjective-effects measurements in assessing abuse liability of drugs in humans. Br J Addict 86: 1563-1570.

4. Chait L, Fischman MW, Schuster CR (1985) 'Hangover' effects the morning after marijuana smoking. Drug and Alcohol Dependence 15: 229-238.

5. Martin W, Sloan J, Sapira J, Jasinski D (1971) Physiologic, subjective, and behavioral effects of amphetamine, methamphetamine, ephedrine, phenmetrazine, and methylphenidate in man. Clin Pharmacol Ther 12: 245-258.

6. Engelhardt BE, Stephens M (2010) Analysis of Population Structure: A Unifying Framework and Novel Methods Based on Sparse Factor Analysis. PLoS Genet 6: e1001117.

7. Korn JM, Kuruvilla FG, McCarroll SA, Wysoker A, Nemesh J, et al. (2008) Integrated genotype calling and association analysis of SNPs, common copy number polymorphisms and rare CNVs. Nature genetics 40: 1253-1260.

8. Carvalho B, Bengtsson H, Speed TP, Irizarry RA (2007) Exploration, normalization, and genotype calls of high-density oligonucleotide SNP array data. Biostatistics 8: 485-499.

9. Purcell S, Neale B, Todd-Brown K, Thomas L, Ferreira MAR, et al. (2007) PLINK: a tool set for whole-genome association and population-based linkage analyses. American Journal of Human Genetics 81: 559-575.

10. Patterson N, Price AL, Reich D (2006) Population structure and eigenanalysis. PLoS Genet 2: e190.

11. Howie BN, Donnelly P, Marchini J (2009) A flexible and accurate genotype imputation method for the next generation of genome-wide association studies. PLoS Genet 5: e1000529.

12. Frazer KA, Ballinger DG, Cox DR, Hinds DA, Stuve LL, et al. (2007) A second generation human haplotype map of over 3.1 million SNPs. Nature 449: 851-861.

13. Durbin RM, Altshuler DL, Abecasis GR, Bentley DR, Chakravarti A, et al. (2010) A map of human genome variation from population-scale sequencing. Nature 467: 1061-1073.

14. Kaiser HF (1958) The varimax criterion for analytic rotation in factor analysis. Psychometrika 23: 187-200.

15. Novembre J, Stephens M (2008) Interpreting principal component analyses of spatial population genetic variation. Nature Genetics 40: 646-649.
